# Sorting of lysosomal enzyme and autophagy are regulated by the GGA1-induced TGN lipid scrambling

**DOI:** 10.1126/sciadv.aec4519

**Published:** 2026-08-28

**Authors:** Kohta Takahashi, Nario Tomishige, Mitsuhiro Abe, Gašper Šolinc, James Rae, Toshiyuki Yamaji, Frédéric Przybilla, Ludovic Richert, Nicolas Humbert, Takefumi Uemura, Thomas Wollert, Satoshi Waguri, Kentaro Hanada, Yasushi Sako, Gregor Anderluh, Robert G. Parton, Yves Mély, Catherine Tomasetto, Fabien Alpy, Toshihide Kobayashi

**Affiliations:** ^1^ Laboratoire de Bioimagerie et Pathologies, UMR 7021 CNRS, Faculté de Pharmacie, Université de Strasbourg, F67401 Illkirch, France.; ^2^ Cellular Informatics Laboratory, RIKEN PRI, Wako, 351-0198 Saitama, Japan.; ^3^ Department of Molecular Biology and Nanobiotechnology, National Institute of Chemistry, Ljubljana 1001, Slovenia.; ^4^ Institute for Molecular Bioscience, The University of Queensland, St. Lucia, Queensland 4072, Australia.; ^5^ Department of Biochemistry and Cell Biology, National Institute of Infectious Diseases. Shinjuku-ku, 162-0052 Tokyo, Japan.; ^6^ Department of Microbiology and Immunology, Faculty of Pharmacy, Juntendo University, Urayasu, 279-0013 Chiba, Japan.; ^7^ Department of Anatomy and Histology, Fukushima Medical University School of Medicine, Fukushima 960-1295, Japan.; ^8^ Laboratoire de Biochimie Membranaire et Transport, UMR 3691 CNRS, Institut Pasteur, 75724 Paris, France.; ^9^ Department of Quality Control, Japan Institute for Health Security (JIHS), Shinjuku-ku, 162-8640 Tokyo, Japan.; ^10^ Centre for Microscopy and Microanalysis, The University of Queensland, St. Lucia, Queensland 4072, Australia.; ^11^ CNRS, Inserm, IGBMC UMR 7104–UMR-S 1258, Université de Strasbourg, F-67400 Illkirch, France.; ^12^ Holo-Bio Co. Ltd., Goryo-Ohara, 615-8245 Kyoto, Japan.; ^13^ Kyoto University of Advanced Sciences, Kameoka, 615-8577 Kyoto, Japan.

## Abstract

The physiological role of lipid asymmetry in intracellular membranes remains poorly understood. Here, we show that sphingomyelin (SM), typically confined to the lumen of the trans-Golgi network (TGN), is exposed on its cytoplasmic surface by the action of the Golgi-associated protein, Golgi-associated gamma-adaptin ear–containing adenosine 5′-diphosphate–ribosylation factor–binding protein 1 (GGA1). This exposure is driven by the GGA1 GAT domain, which induces lipid scrambling in a manner dependent on membrane curvature and cholesterol. SM exposure coincides with the exit of mannose 6-phosphate receptors from the TGN, a process essential for lysosomal enzyme trafficking. Furthermore, SM is transferred to autophagic membranes, where it facilitates autophagosome-lysosome fusion. These findings reveal a previously unrecognized role for lipid remodeling in membrane trafficking and autophagy.

## INTRODUCTION

A hallmark of the mammalian plasma membrane (PM) is its asymmetric lipid distribution. Phosphatidylcholine (PC) is primarily located in the outer (extracellular) leaflet, whereas phosphatidylethanolamine (PE), phosphatidylserine, phosphatidylinositol (PI), and phosphatidylinositol 4,5-bisphosphate (PI(4,5)P_2_) are enriched in the inner (cytoplasmic) leaflet (*1*, *2*). It has been postulated that this asymmetry is established and maintained by flippases, floppases, and scramblases, enzymes that mediate lipid translocation across the bilayer (*3*–*5*).

Sphingomyelin (SM), a major sphingolipid comprising 2 to 15% of total phospholipids in mammalian cells, contains a phosphorylcholine head group and a mainly saturated fatty acid attached to a sphingoid base. In human skin fibroblasts, ∼90% of SM localizes to the PM (*6*), but it is also found in endosomes and the Golgi apparatus. Our current understanding of SM’s transbilayer distribution largely comes from studies on erythrocytes, which contain a single lipid bilayer. Treatment of erythrocytes with bacterial sphingomyelinase (bSMase) led to an ∼85% reduction in SM without hemolysis, indicating that SM is mainly located in the outer leaflet (*7*). As a result, it is widely assumed that SM resides almost exclusively in the outer leaflet of nucleated cell PMs (*1*, *2*, *8*, *9*).

SM is synthesized via two pathways: Bulk SM is produced on the luminal side of the trans-Golgi network (TGN) by sphingomyelin synthases 1 and 2 (SMS1/2) and delivered to the PM via vesicular trafficking (*10*); a local SM pool is synthesized on the extracellular side of the PM by SMS2 (*11*). Thus, the asymmetric distribution of SM is established during its synthesis (*12*).

Contrary to the assumption of strict outer leaflet localization, our previous studies demonstrated that SM is also present in the inner leaflet of the PM in nucleated cells (*13*). However, the mechanisms underlying SM transbilayer movement remain unclear, especially since no known protein catalyzes SM flip-flop. Recent work in our laboratory using SM-binding proteins (SBPs) as probes and immunoelectron microscopy revealed that SM forms distinct lipid domains in the inner leaflet of nucleated cells—but not in erythrocytes (*13*). We also showed that cytosolic overexpression of bSMase led to reduced SM levels in the PM’s outer leaflet, suggesting a dynamic inward transport of SM (*14*).

A genome-wide screen identified candidate proteins that may regulate this asymmetry (*14*). Among them, peripheral myelin protein 2 (PMP2) was found to deform the PM and promote lipid flip-flop. We also identified Golgi-associated gamma-adaptin ear–containing Arf (adenosine 5′-diphosphate–ribosylation factor)–binding protein 1 (GGA1), a Golgi-localized trafficking protein (*14*), which we hypothesized to function at the organelle level.

In this study, we investigated the role of GGA1 in SM translocation. Our findings show that GGA1 promotes SM exposure on the cytoplasmic side of the TGN and induces lipid scrambling in model membranes. GGA1 is known to mediate mannose 6-phosphate receptor (MPR) transport from the TGN to lysosomes (*15*, *16*), facilitating the recruitment of mannose 6-phosphate (M6P)-tagged lysosomal hydrolases (*15*, *17*). We propose that cytoplasmic SM exposure at the TGN is essential for this trafficking pathway. Furthermore, SM is delivered to the phagophore, an initial, cup-shaped membrane structure that plays a critical role in autophagy (*18*), and autophagosomes, where it facilitates autophagosome-lysosome fusion.

## RESULTS

In all experiments except those involving the SMS1/2 double knockout (DKO) mutant, we used HeLa cells from the American Type Culture Collection (ATCC) (CCL-2). The SMS1/2 DKO line was derived from HeLa-mCAT#8 (ectopic receptor protein) cells (*19*, *20*), which stably express the mouse CAT1 receptor. Throughout this study, we refer to HeLa ATCC CCL-2 as wild-type (WT) and HeLa-mCAT#8 as control (Ctrl) cells.

### GGA proteins mediate cytosolic exposure of SM

To visualize SM in the cytoplasmic leaflet of intracellular membranes, we used two SBPs: nontoxic equinatoxin II (NT-EqtII) (*21*) and nontoxic lysenin (NT-Lys) (*22*). EqtII binds dispersed SM, whereas Lys preferentially labels clustered SM (*23*, *24*). Studies using model membranes indicate that Lys does not itself induce SM clustering (*22*).

NT-EqtII-mKate or mCherry-NT-Lys expressed in HeLa cells labeled intracellular structures (fig. S1A). NT-EqtII-mKate was localized to intracellular vesicles as described (*25*). Previously, we showed that cytosolic NT-Lys labels the inner leaflet of the PM in HeLa cells (*14*). Consistent with this, NT-Lys labeled the entire cell and occasionally also intracellular vesicles and the nucleus. Nuclear labeling was not attributable to free mCherry because negligible free mCherry was detected upon mCherry-NT-Lys expression (fig. S1B).

**Fig. 1.**
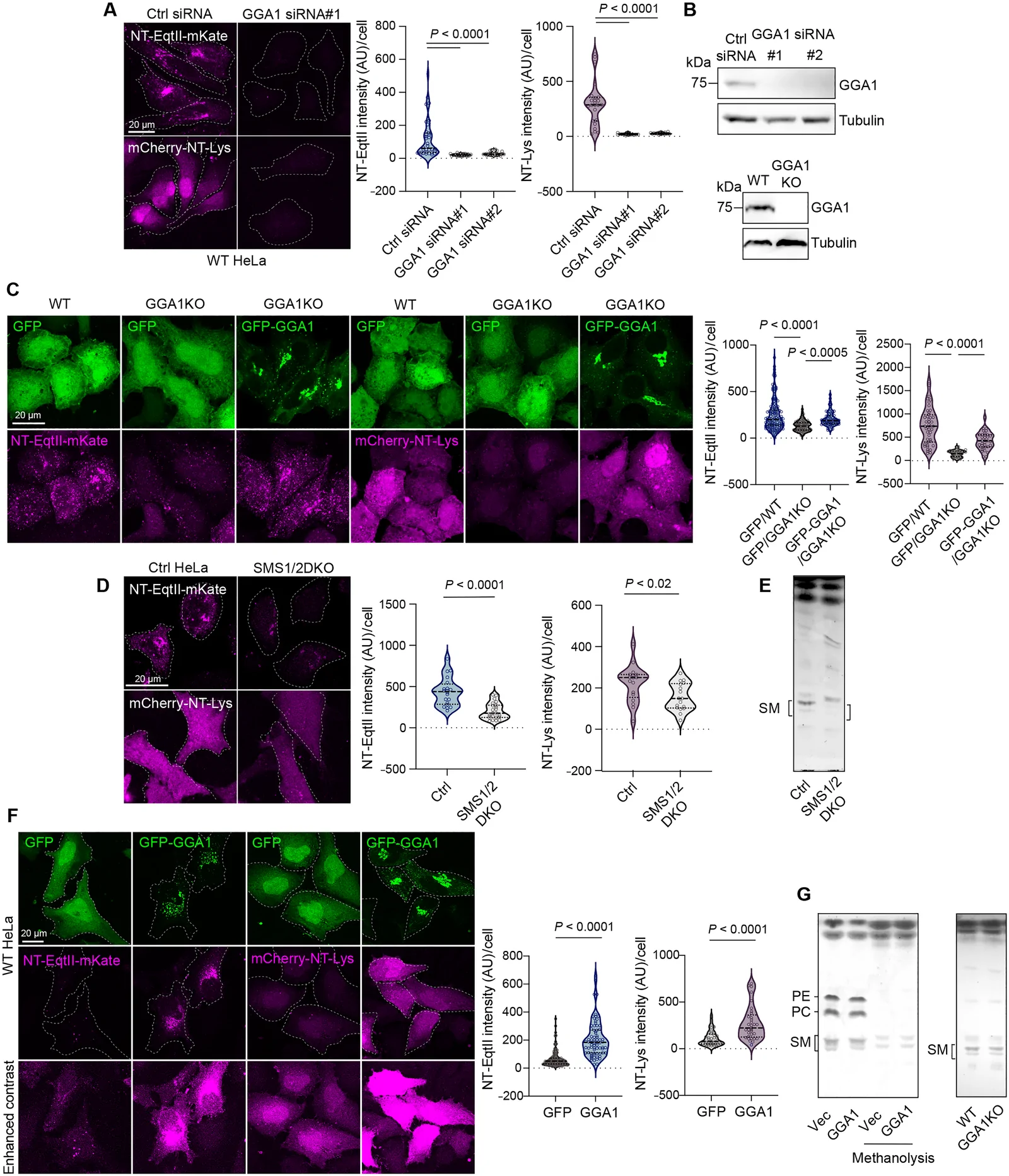
GGA proteins mediate the exposure of SM on the cytosolic leaflet. (**A**) HeLa cells were treated with control or GGA1 siRNA for 72 hours. During the final 24 hours, cells were cotransfected with NT-EqtII-mKate or mCherry-NT-Lys, fixed, and imaged. Fluorescence intensity per cell is shown. *n* = 34 (NT-EqtII-mKate + control), 25 (GGA1 siRNA#1), 23 (GGA1 siRNA#2), 24 (mCherry-NT-Lys + control), 14 (GGA1 siRNA#1) and 17 (GGA1 siRNA#2). AU, arbitrary units. (**B**) GGA1 expression in siRNA-treated (top) and GGA1KO (bottom) cells was assessed by immunoblotting. (**C**) WT and GGA1KO cells were transfected with either GFP or GFP-GGA1 together with NT-EqtII-mKate or mCherry-NT-Lys. Fluorescence intensity per cell was quantified. *n* = 131, 99, 67, 44, 39, and 33 cells, respectively. (**D**) Control and SMS1/2 DKO HeLa cells were transfected with NT-EqtII-mKate or mCherry-NT-Lys, and fluorescence intensity per cell was quantified. The center line represents the median; top and bottom lines denote the 75th and 25th percentiles, respectively. *n* = 20, 23, 17, and 17 cells (NT-EqtII-mKate control, NT-EqtII-mKate DKO, mCherry-NT-Lys control, and mCherry-NT-Lys DKO). (**E**) Total lipids from control and SMS1/2 DKO cells were analyzed by high-performance thin-layer chromatography (HPTLC) following methanolysis. The SM band is indicated. (**F**) WT HeLa cells were transfected with GFP or GFP-GGA1 together with NT-EqtII-mKate or mCherry-NT-Lys. Different contrast settings are shown for the lipid probe images. *n* = 139, 76, 37, and 33 cells, respectively. (**G**) Total lipids from vector control or GGA1 expressing HeLa cells (left), or from WT and GGA1KO cells, were separated by HPTLC before (left) and/or after methanolysis (left and right). Positions of SM, PC, and PE are indicated. All images were acquired by confocal microscopy 24 hours posttransfection. Vec, vector. Statistical analyses: two-tailed Student’s *t* test [(D) and (F)] and one-way analysis of variance (ANOVA) with Tukey’s post hoc test [(A) and (C)]. Data were pooled from at least two independent experiments.

Next, we investigated whether GGA1 regulates cytosolic SM exposure. Small interfering RNA (siRNA)–mediated reduction of GGA1 reduced fluorescence signals from both SM probes (Fig. 1, A and B). Similarly, GGA1 knockout (GGA1KO) cells showed markedly diminished probe signals, which were restored by reexpression of green fluorescent protein (GFP)–GGA1 (Fig. 1, B and C). These findings suggest that GGA1 is responsible for exposing SM to the cytosol.

Unexpectedly, however, GGA1 deficiency did not alter probe localization patterns but instead caused a pronounced overall reduction in fluorescence intensity. To determine whether the loss of SM-probe signals is specific to GGA1 function, we first repeated the staining with SMS1/2DKO mutant (*19*, *20*). SMS1 and SMS2 are the only enzymes that generate SM (*10*, *11*). Similar to GGA1KO cells, SMS1/2 DKO cells exhibited a strong reduction in both NT-EqtII and NT-Lys signals (Fig. 1, D and E).

To exclude the possibility that reduced fluorescence arose from differences in probe expression, we examined probe levels by immunoblotting. NT-EqtII-mKate and mCherry-NT-Lys expression levels were comparable between WT HeLa and GGA1KO, and control HeLa and SMS1/2 DKO cells, and GGA1-silenced cells (fig. S1C).

Although bulk probe expression was similar across conditions, cell-to-cell variability could still potentially affect fluorescence intensity measurements. To address this issue, we implemented a P2A self-cleaving peptide-based coexpression system (*26*). Specifically, we expressed NT-EqtII-mKate2-P2A-mTagBFP2 and mTagBFP2-P2A-mCherry-NT-Lys in GGA1KO cells (fig. S1D), enabling equimolar expression of the lipid probe and BFP within individual cells. We then quantified the fluorescence intensity ratio of each lipid probe relative to BFP, which is expected to distribute uniformly throughout the cytoplasm. These ratios were markedly reduced in GGA1KO cells (fig. S1D), indicating that the observed fluorescence decrease was not caused by altered probe expression.

Binding of soluble NT-EqtII-mKate and mCherry-NT-Lys to membrane SM markedly increases their local concentration by restricting diffusion from three dimensions to two dimensions (*27*, *28*). In addition, SM alone or together with glycosphingolipids forms specific lipid domains that further concentrate these probes locally (*29*, *30*). Thus, the marked reduction in fluorescence intensity observed in GGA1KO and SMS1/2 DKO cells is consistent with a redistribution of the probes from membrane-associated [two-dimensional (2D)] to freely diffusing cytosolic (3D) states.

To further validate probe performance under conditions of altered SM accessibility, we treated cells with l-leucyl–l-leucine O-methyl ester (LLOMe), which disrupts lysosomes and exposes luminal SM to the cytosol. Under these conditions, NT-EqtII labeled punctate structures (fig. S1E), consistent with previous observations using the related EqtSM probe (*31*).

Overexpressed GFP-GGA1 localized to vesicular and tubular structures that partially overlapped with the TGN marker, mKate-β-1,4-galactosyltransferase (fig. S1F and movie S1). Compared with GFP-expressing control cells, GFP-GGA1–expressing cells exhibited markedly increased intracellular labeling by both NT-EqtII-mKate and mCherry-NT-Lys (Fig. 1F). Despite these changes in probe labeling, total cellular SM levels remained unchanged following GGA1 overexpression or depletion (Fig. 1G), suggesting that GGA1 regulates SM exposure rather than SM synthesis. Probe expression levels also remained comparable among conditions (fig. S1C).

To further control for expression variability, we expressed NT-EqtII-mKate2-P2A-mTagBFP2 and mTagBFP2-P2A-mCherry-NT-Lys in GGA1-overexpressing cells (fig. S1G). In these cells, the fluorescence intensity ratios of both lipid probes relative to BFP were significantly increased.

We next quantified the organelle-to-cytosol distribution of NT-EqtII fluorescence in confocal single optical sections (fig. S1H). NT-EqtII predominantly colocalized with LAMP1-positive compartments (see below). The fraction of NT-EqtII fluorescence associated with LAMP1-positive structures relative to cytosolic fluorescence was markedly increased in GFP-GGA1–expressing cells. This observation supports the interpretation that increased probe intensity reflects local enrichment on membrane structures rather than increased total probe abundance.

Together, these results indicate that GGA1 facilitates the exposure of SM on the cytoplasmic leaflet of intracellular membranes.

GGA1 belongs to the GGA protein family, which also includes GGA2 and GGA3; all three proteins share similar structural features (*15*). Reported mRNA abundances in HeLa cells are 38.6, 25.3, and 15.0 transcripts per million for GGA1, GGA2, and GGA3, respectively (*32*). Overexpression of either GGA2 or GGA3 similarly enhanced labeling by NT-EqtII-mKate and mCherry-NT-Lys (fig. S1, I and J), suggesting that all three GGA proteins promote cytosolic SM exposure. Because GGA proteins form complexes on Golgi membranes (*33*), the reduction of GGA1 may disrupt formation of the functional GGA complex. Collectively, these findings demonstrate that GGA proteins, particularly GGA1, are required for proper exposure of SM on the cytoplasmic leaflet of intracellular membranes.

### The GAT domain of GGA1 promotes lipid scrambling

GGA1 has a modular structure comprising three conserved domains: VHS (Vps27, Hrs, and STAM), GAT (GGA and TOM1), and GAE (gamma-adaptin ear) (*15*). The VHS domain mediates binding to the MPR and other cargo proteins and also interacts with phosphatidylinositol 4-phosphate (PI4P) (*15*, *34*). The GAT domain binds PI4P, ubiquitin, and the small guanosine triphosphatase (GTPase) Arf, while the GAE domain associates with various accessory proteins (*15*).

**Fig. 2.**
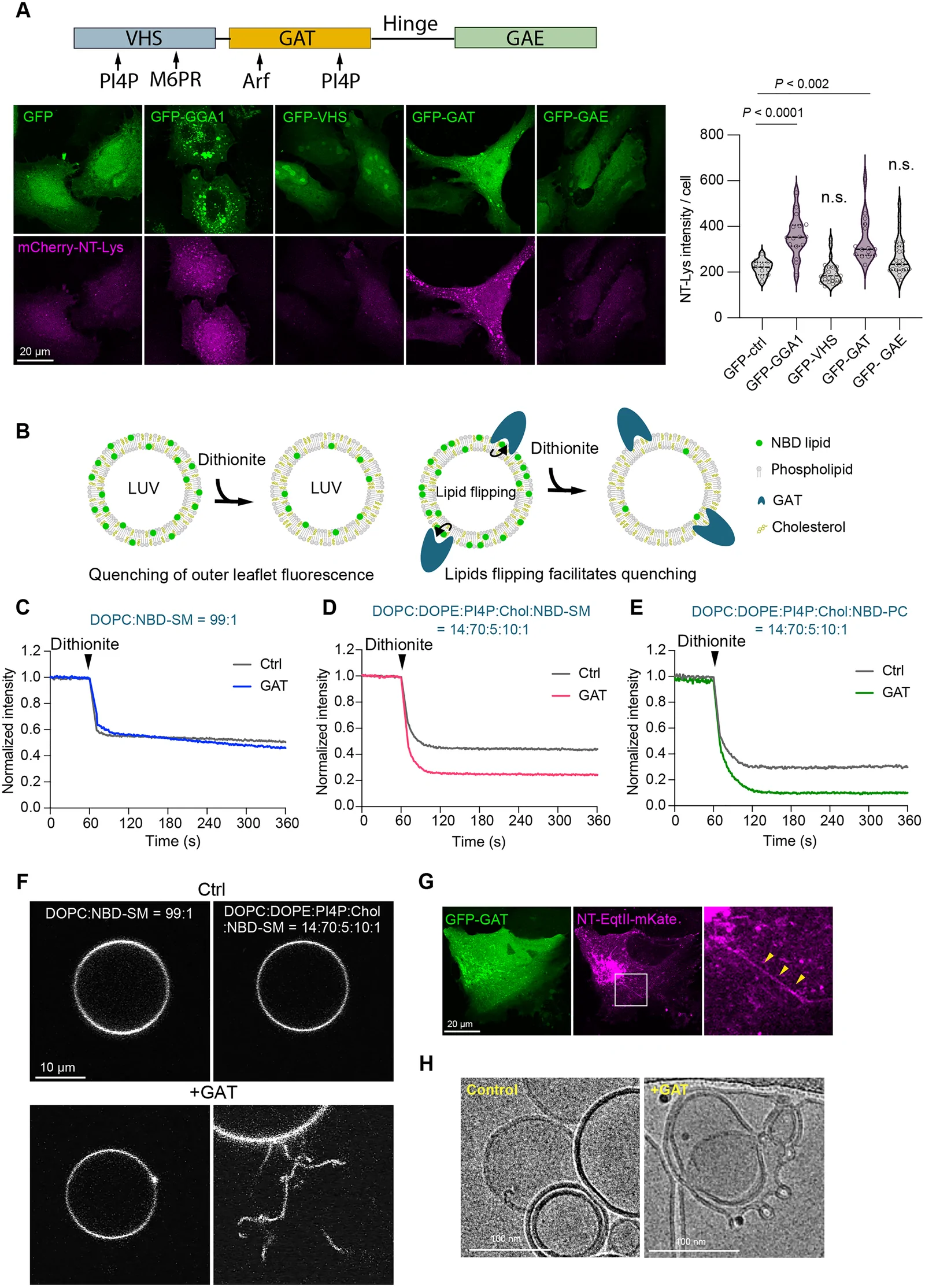
The GAT domain of GGA1 promotes lipid scrambling. (**A**) Top left: Schematic of GGA1 structure. GGA1 contains VHS, GAT, and GAE domains. The VHS domain binds MPR and PI4P; the GAT domain interacts with Arf and PI4P. Bottom left: WT HeLa cells were cotransfected with mCherry-NT-Lys and GFP, GFP-GGA1, GFP-VHS, GFP-GAT, or GFP-GAE. Confocal images were acquired 24 hours posttransfection. Right: Fluorescence intensity of mCherry-NT-Lys per cell was quantified. *n* = 11 (GFP), 16 (GFP-GGA1), 25 (GFP-VHS), 15 (GFP-GAT), and 25 (GFP-GAE). One-way ANOVA with Tukey’s post hoc tests. (**B**) Schematic of the dithionite assay to assess interleaflet distribution of NBD-labeled lipids. NBD lipids are incorporated into both leaflets of LUVs, but only outer leaflet NBDs are quenched by sodium dithionite. Lipid flipping from the inner to outer leaflet increases quenching, which serves as a readout for translocation. (**C** to **E**) LUVs composed of (C) DOPC:NBD-SM (99:1), (D) DOPC:DOPE:PI4P:Chol:NBD-SM (14:70:5:10:1), and (E) DOPC:DOPE:PI4P:Chol:NBD-PC (14:70:5:10:1) and were incubated with or without purified 1 μM GAT protein. Sodium dithionite was added at 60 s during NBD fluorescence measurement. Predithionite fluorescence was set to 1; relative fluorescence was calculated. Data represent mean values from three to four independent experiments. (**F**) Giant unilamellar vesicles (GUVs) composed of DOPC:NBD-SM (99:1) or DOPC:DOPE:PI4P:Chol:NBD-SM (14:70:5:10:1) were incubated ±1 μM GAT protein for 30 min at room temperature (RT). Images were acquired postincubation. See also movie S2. (**G**) WT HeLa cells were cotransfected with GFP-GAT and NT-EqtII-mKate. Confocal images were acquired 24 hours posttransfection. The region outlined by the white square in the central panel is shown at higher magnification in the right panel. Arrowheads indicate NT-EqtII-mKate–positive tubules. See also movie S3. (**H**) Cryo–electron micrographs of LUVs incubated with or without GAT protein. Imaging was performed as described in Materials and Methods.

To identify which domain promotes SM exposure to the cytosolic leaflet, we coexpressed GFP-tagged versions of each domain with mCherry-NT-Lys in HeLa cells. Only the GAT domain induced strong NT-Lys labeling mimicking GGA1, suggesting its key role in SM translocation (Fig. 2A).

To determine whether this is a direct property of the GAT domain, we purified the recombinant GAT domain (fig. S2A) and used a model membrane liposome system (see schemes in Fig. 2B). Large unilamellar vesicles (LUVs) doped with 1% fluorescent lipid, *N*-[12-[(7-nitro-2-1,3-benzoxadiazol-4-yl)amino]dodecanoyl]-sphingosine-1-phosphocholine (NBD-SM), were incubated with the GAT domain of GGA1. In this assay, liposomes are treated with sodium dithionite, an irreversible quencher of NBD that penetrates membranes very slowly. Consequently, only the fluorescence of NBD on the outer leaflet is initially quenched (*35*). Any exposure of inner leaflet NBD-SM to the quencher, due to transbilayer movement, would result in further reduction of total NBD fluorescence associated with the liposomes. In simple dioleoylphosphatidylcholine (DOPC):NBD-SM (99:1) liposomes, GAT did not induce SM flipping (Fig. 2C).

Because GGA1 is a cytosolic protein, it interacts with the TGN from the cytoplasmic side of the organelle. Given that the Golgi transbilayer lipid distribution is not well understood, we assumed that it has a similar lipid asymmetry to the PM. In the PM, PE is exclusively distributed in the cytosolic leaflet and PC to the exoplasmic leaflet (*1*, *13*). Accordingly, we prepared PE-rich liposomes representing the cytosolic leaflet of the Golgi apparatus and included PI4P, the main phosphoinositide of the TGN known to bind the GAT domain (*34*). We also added cholesterol (Chol) to the liposomes.

When liposomes composed of DOPC:DOPE:brain PI4P:Chol:NBD-SM (14:70:5:10:1) were treated with sodium dithionite, a similar reduction in fluorescence (∼50%) was observed as with DOPC:SM (99:1), showing symmetrical distribution of SM in LUVs. Notably, when these liposomes were incubated with the GAT domain, the fluorescence intensity decreased by 75% (Fig. 2D), suggesting that the GAT domain induces transbilayer movement of NBD-SM. The presence of GAT did not completely eliminate NBD fluorescence in the dithionite assay.

Further addition of GAT did not cause additional decrease of NBD fluorescence (fig. S2B), indicating that the quenching response had reached a plateau under our experimental conditions. Cryo–electron microscopy (cryo-EM) images of the liposomes used in these experiments are shown in fig. S2C. In addition to monolamellar liposomes, double-lamellar liposomes were detected. Of the 49 liposomes analyzed, 15 exhibited a double-lamellar structure, and a population of very small liposomes was also present. These small liposomes are expected to have an outer-to-inner lipid ratio greater than 1. This structural heterogeneity likely contributes to the incomplete quenching observed in the presence of GAT, whereas ∼50% or more of the fluorescence was quenched in its absence.

To test whether the GAT domain is able to permeabilize membranes, we performed collisional quenching of NBD fluorescence with potassium iodide (KI) (fig. S2D) (*36*). NBD fluorescence was quenched to a similar extent in the absence and presence of GAT, indicating that GAT does not permeabilize the membrane. Consistently, a calcein leakage assay (*37*) showed that incubation with GAT did not increase calcein release compared to the control (fig. S2E). Together, these results demonstrate that GAT does not compromise membrane integrity.

To better understand the structural determinant implicated in the function of GAT, we generated *N*-hydroxysuccinimide (NHS)–Cy3–labeled GAT (NHS-Cy3-GAT), in which lysine residues were chemically modified. In contrast to unmodified GAT, NHS-Cy3-GAT failed to induce transbilayer movement of NBD-SM (fig. S2F), indicating that lysine residues are critical for GAT-mediated lipid flip-flop. We next assessed the transbilayer movement of NBD-PC and found that, similar to NBD-SM, the GAT domain induced efficient transbilayer movement of NBD-PC (Fig. 2E). Together, these results indicate that the GAT domain of GGA1 promotes lipid scrambling in model membranes.

GGA1 is not a transmembrane but a cytosolic protein, raising the question of how it induces transbilayer lipid movement. Previously, we demonstrated that another cytosolic protein, PMP2, promotes transbilayer movement of SM in the PM and LUV and induces membrane tubulation in giant unilamellar vesicles (GUVs) (*14*). Similarly, the GAT domain induced pronounced membrane tubulation in GUVs composed of DOPC:DOPE:PI4P:Chol:NBD-SM (Fig. 2F and movie S2), whereas this effect was not observed in DOPC:NBD-SM GUVs. Moreover, tetramethyl rhodamine isothiocyanate (TRITC)–dextran [molecular weight (MW): 10,000] added to the external medium did not enter GUVs either in the absence or presence of GAT (fig. S2G), confirming that GAT did not permeabilize liposomes.

In vitro experiments show that the GAT domain of GGA1 promotes SM flipping and membrane tubulation in liposomes. To investigate whether the GAT domain has a similar function in cells, we performed live imaging of cells expressing the GFP-tagged GAT domain along with the SM-probe NT-EqtII-mKate (Fig. 2G and movie S3). Overexpression of the GFP-GAT induced the formation of tubules labeled by NT-EqtII-mKate (Fig. 2G and movie S3). These tubules were derived from the GAT-enriched compartments. However, GFP-GAT and NT-EqtII-mKate did not colocalize, suggesting that the GAT domain either does not associate with the membrane tubules or interacts with them only transiently. Cryo-EM analysis of LUVs revealed budding structures induced by the GAT domain (Fig. 2H and fig. S2H). The shorter buds observed in LUVs, compared to the longer tubules seen in GUVs, may result from the size difference between the liposomes. We speculate that the excess membranes in GUVs allow for the extended formation of buds. These results indicate that the GAT domain of GGA1 promotes lipid scrambling in model membranes and induces tubulation in both cell and model membranes.

### Chol regulates GGA1-mediated SM flipping

**Fig. 3.**
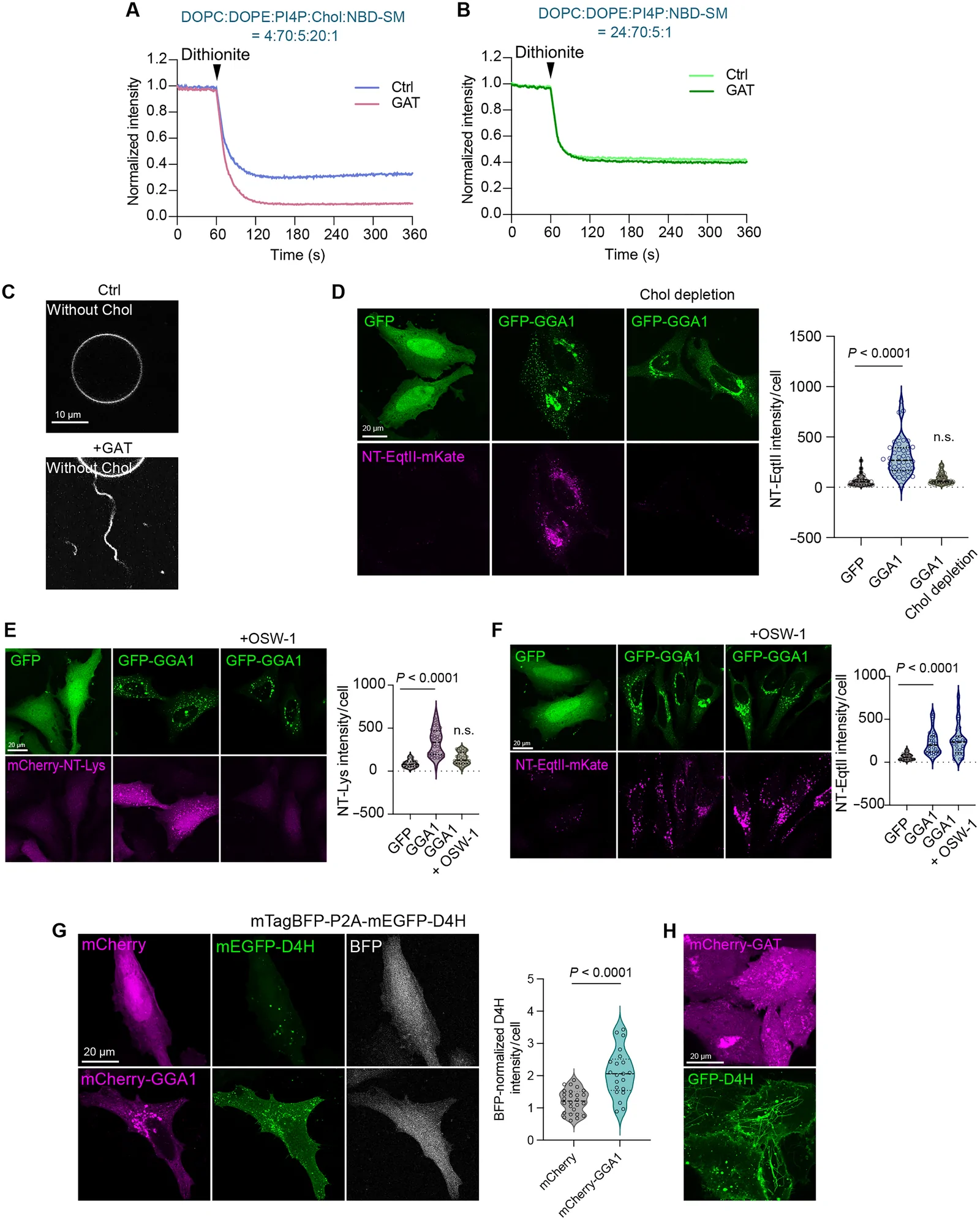
Chol regulates the transbilayer movement of SM. (**A** and **B**) LUVs composed of (A) DOPC:DOPE:PI4P:Chol:NBD-SM (4:70:5:20:1) and (B) DOPC:DOPE:PI4P:NBD-SM (24:70:5:1) were incubated with or without purified 1 μM GAT protein. Dithionite treatment and fluorescence analysis were performed as described in Fig. 2 (C to E). *n* = 3 to 4. (**C**) GUVs composed of DOPC:DOPE:PI4P:NBD-SM (24:70:5:1) were incubated ±1 μM GAT protein. Images were acquired as described in Fig. 2F. See also movie S4. (**D**) WT HeLa cells were cotransfected with GFP + NT-EqtII-mKate or GFP-GGA1 + NT-EqtII-mKate. To deplete Chol, a subset was treated with 100 μM lovastatin and 10 μM mevalonate in 5% lipoprotein-deficient serum (LPDS). Confocal images were acquired 24 hours posttransfection. NT-EqtII-mKate fluorescence per cell was quantified. *n* = 39 (GFP), 40 (GFP-GGA1), and 41 (GFP-GGA1 + Chol depletion). n.s., not significant. (**E**) WT cells were cotransfected with GFP + mCherry-NT-Lys or GFP-GGA1 + mCherry-NT-Lys. A subset was treated with 20 nM OSW-1 for the final 4 hours. Images were acquired after 24 hours. mCherry-NT-Lys fluorescence was quantified. *n* = 35 (GFP), 38 (GFP-GGA1), and 32 (GFP-GGA1 + OSW-1). (**F**) Cells were transfected as in (E) but with NT-EqtII-mKate. NT-EqtII-mKate fluorescence was quantified. *n* = 32 (GFP), 32 (GFP-GGA1), and 28 (GFP-GGA1 + OSW-1). (**G**) WT cells were cotransfected with mTagBFP2-P2A-mEGFP-D4H + mCherry or mCherry-GGA1. Confocal images were acquired in live cells. GFP/BFP ratios were quantified. *n* = 26 (mCherry) and 21 (mCherry-GGA1). (**H**) WT HeLa cells were cotransfected with mCherry-GAT and GFP-D4H. Confocal images were acquired 24 hours posttransfection in live. See also movie S5. Statistical analyses: two-tailed Student’s *t* test (G) and one-way ANOVA with Tukey’s post hoc test [(D) to (F)]. Data were pooled at least from two independent experiments.

Having established a direct function of the GAT domain on SM flipping and membrane tubulation, we next investigated the lipid requirements for this function. Using an in vitro approach, we modified the lipid composition of liposomes and measured SM flipping in the presence of the GAT domain of GGA1. Reducing DOPE or removing Chol abolished GAT-mediated SM flipping (Fig. 3, A and B, and fig. S2, I to K). In GUVs lacking Chol, tubulation still occurred (Fig. 3C and movie S4), indicating that while tubulation does not require Chol, lipid flipping does.

We then extended this assay to cells by depleting Chol. Cellular Chol depletion was achieved by removing Chol from the medium using lipoprotein-deficient serum (LPDS) and by inhibiting Chol biosynthesis with lovastatin treatment (*38*). The reduction in cellular Chol was confirmed by labeling cellular free Chol using a GFP-tagged Chol-specific probe, D4H, after fixation and permeabilization. This probe is derived from the Chol-binding D4 domain of perfringolysin O and has a lower concentration threshold for Chol binding than D4 (fig. S2L) (*39*–*41*). Notably, GGA1-induced SM exposure was abolished upon Chol depletion, indicating that the protein promotes SM exposure specifically in Chol-containing membranes (Fig. 3D).

Next, we used a more specific approach to reduce Chol on the Golgi by inhibiting oxysterol binding protein (OSBP), which exchanges Chol for PI4P between the Golgi apparatus and the endoplasmic reticulum (ER) (*42*, *43*). In cells expressing GGA1, blocking OSBP-mediated Chol transfer with OSW-1 (*44*) selectively reduced NT-Lys (clustered SM) labeling, while NT-EqtII (dispersed SM) remained largely unaffected (Fig. 3, E and F). This suggests that OSW-1 treatment reduces SM clusters without affecting SM monomers (*24*). These findings indicate that OSBP-mediated Chol transport contributes to GGA1-dependent SM flipping.

In addition, the expression of GGA1 protein or its GAT domain in isolation increased the D4H-accessible pool of cytoplasmic Chol, as shown by enhanced labeling of vesicles and tubules by the fluorescent probe (Fig. 3, G and H, and movie S5). These results suggest that GGA1 acts in a Chol-dependent manner to promote the cytosolic exposure of SM.

### The GAT domain of GGA1 forms trimers

Model membrane and cellular studies indicate that the GAT domain promotes membrane tubulation. However, the interaction between the GAT domain and membranes appears to be transient in cells. To better understand this, we further investigated the interaction between the GAT domain and lipids. A protein-lipid overlay assay showed that GAT binds several negatively charged lipids (e.g., PI4P), but not SM or Chol (fig. S3A). However, in liposome-binding assays, GAT did not stably associate with DOPC:DOPE:PI4P:Chol:NBD-SM (14:70:5:10:1) liposomes (fig. S3B), and PI4P removal had no effect on SM flipping (fig. S3C).

Mass photometry suggested that a portion of the GAT domain exists as trimers in solution (fig. S3D). Cryo-EM analysis revealed that some LUVs were surrounded by numerous small vesicles, often associated with protein assemblies (fig. S3E). 2D classification demonstrated that the smallest organizational unit of the protein assemblies is the GAT domain trimers (fig. S3, F and G, and movie S6). The class averages matched the predicted structure of the trimeric GAT domain as modeled by AlphaFold 3 (fig. S3H) (*45*). These findings suggest that GAT interacts with membranes as trimers.

### GGA1-induced SM exposure requires PI4P in cells

Although the GAT domain promotes lipid scrambling in liposomes without strong membrane association, GGA1 induces SM exposure in specific cellular membranes, suggesting that membrane targeting is crucial for GGA1 function. GGA1 is recruited to the TGN via PI4P and the small GTPase, Arf1 (*34*, *46*). We therefore investigated the roles of PI4P and Arf1 in SM exposure. Compared to the WT GAT domain, the PI4P-binding–deficient GAT mutant Arg260→Ala (R260A) induced significantly less SM exposure to the cytosolic leaflet in HeLa cells (fig. S4A). In contrast, full-length GGA1-R260A still promoted SM exposure, but SM was accumulated in the TGN (fig. S4B). This exposure is likely due to residual PI4P binding via the VHS domain (*34*).

Treatment with phosphatidylinositol 4-kinase (PI4K) inhibitors—2-(3-(4-chlorobenzoyl)thioureido)-4-ethyl-5-methylthiophene-3-carboxamide (PI-273) (*47*) or phenylarsine oxide (*48*)—blocked GGA1 recruitment to the TGN and suppressed SM exposure (fig. S4C), consistent with PI4P-dependent membrane targeting of GGA1 (*34*).

We then examined the effect of Arf1 on SM exposure. The activated form of Arf1 [Arf1-guanosine 5′-triphosphate (GTP)] interacts with the N-terminal region of the GAT domain of GGA1 (*46*). Overexpression of GFP-Arf1, its constitutively active mutant (GFP-Arf1 Gln71→Ala (Q71L)-HA; GTP-bound form), or its inactive mutant (GFP-Arf1 Thr31→Asn (T31N)-HA; guanosine diphosphate–bound form) had no effect on SM exposure (fig. S4D), suggesting that Arf1 is dispensable for this process. Together, these findings indicate that GGA1-induced SM exposure in cells is dependent on PI4P-mediated recruitment to the TGN but not on Arf1 binding.

### SM exposure to the cytoplasmic leaflet regulates lysosomal enzyme sorting

**Fig. 4.**
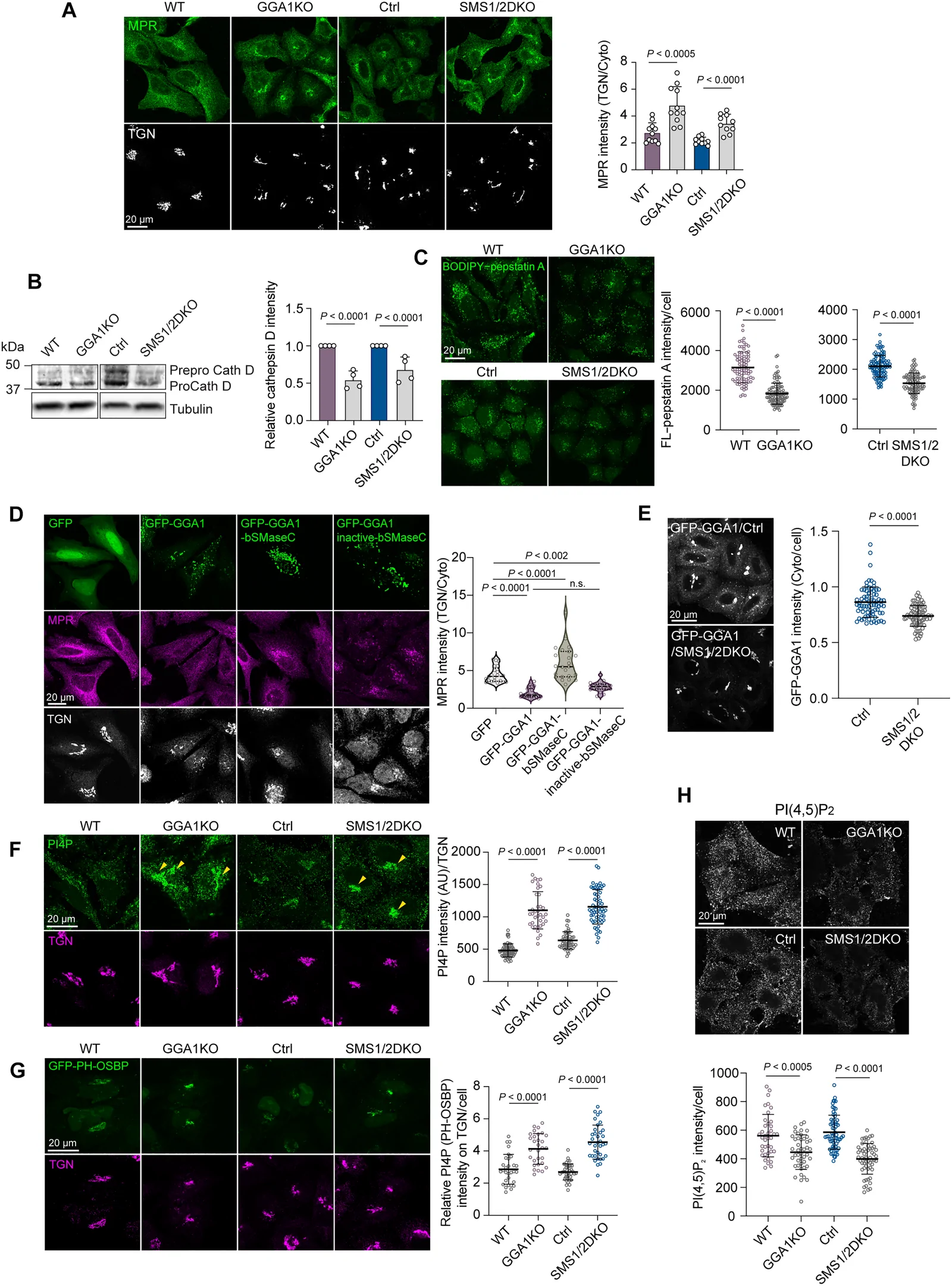
SM exposure to the cytoplasmic leaflet regulates lysosomal enzyme sorting. (**A**) Cells were labeled for CI-MPR and TGN46. The ratio of CI-MPR fluorescence intensity in the TGN to cytoplasmic vesicles (Cyto) was quantified per cell. Mean ± SD. *n* = 11 (WT), 10 (GGA1KO), 10 (control), and 10 (SMS1/2 DKO). (**B**) Representative immunoblot of cathepsin D. Positions of prepro- and pro–cathepsin D were estimated by MW. Relative pro–cathepsin D levels were quantified and normalized to WT or control. Mean ± SD; *n* = 4. (**C**) Cells were incubated with 1 μM BODIPY–pepstatin A for 1 hour at 37°C, fixed, and imaged. Cellular fluorescence (FL) was quantified. Mean ± SD. *n* = 83 (WT), 102 (GGA1KO), 107 (control), and 95 (SMS1/2 DKO). Cath D, cathepsin D. (**D**) WT cells expressing GFP, GFP-GGA1, GFP-GGA1-bSMaseC, or its inactive mutant were labeled for CI-MPR and TGN46. The TGN-to-vesicle CI-MPR fluorescence ratio was quantified. *n* = 21 to 23 cells per condition. (**E**) GFP-GGA1 was expressed in control and SMS1/2 DKO cells. The ratio of vesicular to total GFP-GGA1 fluorescence was quantified. Mean ± SD. *n* = 77 per condition. (**F**) PI4P and TGN were labeled using specific antibodies. PI4P fluorescence in the TGN was quantified. Mean ± SD. *n* = 36 to 64 cells. (**G**) PI4P distribution was assessed using the GFP–PH-OSBP probe. The TGN-to-total GFP fluorescence ratio was quantified. Mean ± SD. *n* = 30 to 40 cells. (**H**) PI(4,5)P_2_ was labeled and quantified per cell. Mean ± SD. *n* = 39 to 67 cells. Statistical analyses: paired (B) or two-tailed Student’s *t* test [(A), (C), and (E) to (H)] and one-way ANOVA with Tukey’s post hoc test (D). Data were pooled from at least two independent experiments.

We next investigated the relationship between SM exposure and a well-established physiological role of GGA proteins, namely, the trafficking of lysosomal hydrolases via the MPR pathway (*15*). In HeLa cells, cation-independent mannose 6-phosphate receptor (CI-MPR) localized to the TGN and dispersed endosomes (Fig. 4A) (*49*). In both GGA1KO and SM-deficient (SMS1/2 DKO) cells, CI-MPR accumulated in the TGN (Fig. 4A), indicating defective TGN-to-endosome transport.

In addition, pro–cathepsin D levels were reduced in these cells (Fig. 4B), consistent with missorting and increased secretion (*33*). Live-cell labeling with BODIPY–pepstatin A, a marker of active lysosomal cathepsin D (*50*), showed reduced signal in both KO models (Fig. 4C), supporting a role for SM in proper hydrolase trafficking.

We also examined whether SM exposure affects the TGN-to-PM transport using an enhanced GFP (EGFP)–tagged vesicular stomatitis virus (VSV)–G protein ts045 mutant. This temperature-sensitive mutant accumulates in the TGN at 20°C. Shifting the temperature to 37°C releases the protein, allowing its transport to the PM (*51*, *52*). VSV-G was efficiently transported to the PM in both GGA1KO and WT cells (fig. S5A).

We further assessed protein secretion using the Retention Using Selective Hooks (RUSH) system (*53*) by expressing the Str-KDEL-SS-SBP-MMP11-EGFP construct, which enables synchronized release of matrix metalloproteinase 11 (MMP11) (*54*) from the ER and its exit from the TGN. Both WT and GGA1KO cells exhibited comparable kinetics of MMP11 transport (fig. S5B and movie S7). Together, these results indicate that depletion of GGA1 does not impair vesicular transport from the TGN to the PM. This is consistent with our previous observation that SM in the outer leaflet of PM was increased in GGA1 knockdown cells (*14*).

To directly assess the role of cytosolic SM on CI-MPR transport, we expressed a GGA1-bSMaseC fusion protein, which degrades SM exposed to the cytoplasmic leaflet. This resulted in loss of NT-EqtII-mKate fluorescence, indicating SM degradation (fig. S6A), and led to the accumulation of CI-MPR at the TGN (Fig. 4D). In contrast, expression of WT GGA1 or a fusion between GGA1 and the catalytically inactive bSMaseC mutant [Asp321→Ala and His322→Ala (D321A/H322A)] (*55*) did not promote the accumulation of CI-MPR at the TGN.

These results demonstrate that GGA1-mediated exposure of SM to the cytoplasmic leaflet is essential for lysosomal hydrolase trafficking via the CI-MPR pathway.

### SM exposure regulates PI4P metabolism and the exit of GGA1-containing transport carriers from the TGN

GGA1 mediates lysosomal enzyme trafficking by exiting the TGN together with CI-MPR (*56*). Our results suggest that SM exposure plays a role in GGA1’s exit from the TGN. To assess whether SM exposure affects GGA1 trafficking, we examined GFP-GGA1 localization in control and SMS1/2 DKO cells. In control cells, GFP-GGA1 localized to the TGN and small cytoplasmic vesicles. In contrast, the number of cytoplasmic GFP-GGA1 vesicles was markedly reduced in SMS1/2 DKO cells (Fig. 4E), suggesting impaired exit from the TGN.

Total GFP-GGA1 fluorescence was also decreased in SMS1/2 DKO cells, despite unchanged protein levels (fig. S6B), probably due to loss of membrane association that enhances fluorescence signal, as seen with lipid-binding probes. These findings indicate that SM is required for both GGA1 recruitment to the TGN and its subsequent vesicular trafficking.

Formation of PI(4,5)P_2_ has been proposed to be associated with membrane vesicle budding (*57*). PI(4,5)P_2_ synthesis from PI4P by PI4P 5-kinase is Arf1 dependent (*58*) and also regulates Arf1–GTPase activating protein (GAP) activity (*59*). We therefore investigated whether SM exposure affects PI4P/PI(4,5)P_2_ metabolism.

Using PI4P-specific monoclonal antibodies (*60*), we observed that both GGA1KO and SMS1/2 DKO cells show increased PI4P accumulation in the TGN compared to controls (Fig. 4F, arrowheads). Similar results were obtained using the PI4P probe, PH-OSBP (Fig. 4G). Conversely, PI(4,5)P_2_ levels on endomembranes were markedly reduced in both KO cell types (Fig. 4H). These alterations were rescued by GGA1 overexpression (fig. S6C), suggesting a regulatory role for GGA1 and SM in phosphoinositide metabolism.

To test whether cytoplasmic SM regulates PI4P distribution, we overexpressed GFP-GGA1-bSMaseC. In WT cells, GFP-GGA1 overexpression dispersed PI4P throughout the cytoplasm, while GFP-GGA1-bSMaseC did not alter PI4P distribution, which remained confined to the perinuclear region, similar to GFP alone (fig. S6D, arrowheads). The catalytically inactive mutant behaved similar to WT GGA1, confirming that cytosolic SM regulates PI4P localization.

The PI4P-binding–deficient GAT mutant GGA1 R260A did not block SM exposure (fig. S4B) yet caused CI-MPR and PI4P to accumulate in the perinuclear region (fig. S6, E and F, arrowheads). These results demonstrate that PI4P binding through the GAT domain is essential for proper PI4P turnover and CI-MPR exit from the TGN. They also suggest that cytoplasmic SM cooperates with GGA1 in regulating PI4P turnover and CI-MPR exit.

### Exposed SM is transported to phagophores, autophagosomes, and late endosomes and lysosomes

**Fig. 5.**
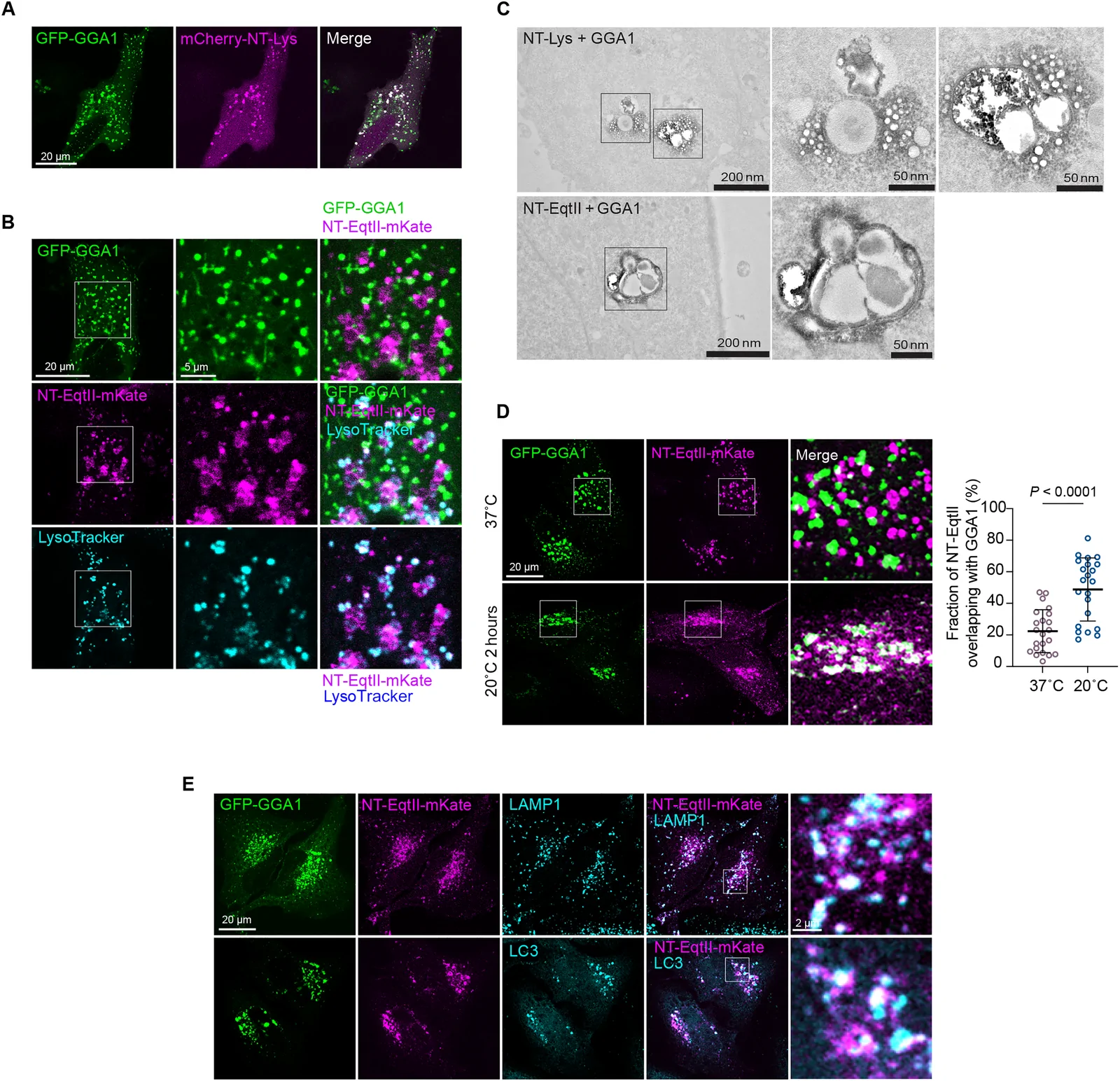
Exposed SM is transported to phagophores, autophagosomes, and late endosomes and lysosomes. (**A**) WT HeLa cells were transfected with GFP-GGA1 and mCherry-NT-Lys. Confocal images were acquired 24 hours posttransfection. (**B**) WT HeLa cells were cotransfected with GFP-GGA1 and NT-EqtII-mKate and treated with LysoTracker Deep Red during the final 30 min. Confocal images were acquired 24 hours posttransfection. (**C**) APEX EM detection of SM in cells overexpressing either EGFP-NT-Lys (top) or NT-EqtII-mClover3 (bottom), coexpressed with mCherry-GGA1 and detected using the APEX2-GBP construct. (**D**) WT HeLa cells were cotransfected with GFP-GGA1 and NT-EqtII-mKate. Cells were incubated either for 24 hours at 37°C or for 22 hours at 37°C, followed by 2 hours at 20°C. Confocal images were acquired postfixation. Right: The proportion of NT-EqtII-mKate fluorescence overlapping with GFP-GGA1 was quantified. Mean ± SD. *n* = 22 cells. (**E**) WT HeLa cells were cotransfected with GFP-GGA1 and NT-EqtII-mKate. After 24 hours, cells were fixed, permeabilized, and immunolabeled with antibodies against LAMP1 (top) or LC3 (bottom). Statistical analyses: two-tailed Student’s *t* test (D), pooled from two independent experiments.

Using SM-specific probes, we found that cytoplasmically exposed SM was present not only at the TGN but also at the PM, nuclear area (both nuclear membrane and matrix), and intracellular vesicles in GGA1-overexpressing cells. This suggests that cytoplasmically exposed SM is transported from the TGN to various destinations (fig. S7A). In live cells, NT-Lys colocalized with GGA1-positive and other unidentified vesicles (Fig. 5A and movie S8). In contrast, NT-EqtII primarily labeled LysoTracker-positive late endosomes and lysosomes and other vesicular structures, with minimal colocalization with GGA1 (Fig. 5B, fig. S7A, and movie S9). EM showed that both NT-Lys and NT-EqtII labeled irregular vesicles containing undegraded material, suggesting the labeling of phagophores and autophagosomes (Fig. 5C and fig. S7B).

Despite both GGA1 and EqtII localizing to vesicles, their minimal colocalization suggests distinct trafficking pathways to late endosomes/lysosomes. To further investigate the EqtII-labeled SM transport route, we incubated HeLa cells at 20°C for 2 hours, a condition that blocks TGN-derived vesicle trafficking (*61*). Under these conditions, NT-EqtII-mKate accumulated in the perinuclear region and colocalized with GGA1 (Fig. 5D), suggesting that SM is transported to endolysosomes via TGN-derived vesicles. Moreover, the number of peripheral NT-EqtII–positive vesicles decreased at 20°C, indicating that SM in endolysosomes turns over within 2 hours. Notably, in cells expressing GGA1-R260A, NT-EqtII-mKate accumulated at the TGN, unlike in cells expressing WT GGA1 (fig. S4B), indicating that PI4P binding via the GAT domain is required for SM exit from the TGN.

To further characterize NT-EqtII–positive vesicles, we coexpressed NT-EqtII-mKate with LAMP1 (*62*, *63*) or LC3 (*64*), markers of late endosomes/lysosomes and phagophores and autophagosomes, respectively. NT-EqtII partially colocalized with both markers (Fig. 5E), consistent with the presence of SM in both compartments. Flow cytometry of autophagic vesicle fraction confirmed enrichment of the NT-EqtII signal in WT cells, but not GGA1KO cells (fig. S7C). In contrast, CI-MPR did not localize to autophagosomes (fig. S7D), suggesting segregation of SM and MPR following TGN export. These results indicate that SM and MPR are transported to late endosomes and lysosomes via distinct routes.

### Cytoplasmic SM regulates autophagosome-lysosome fusion

**Fig. 6.**
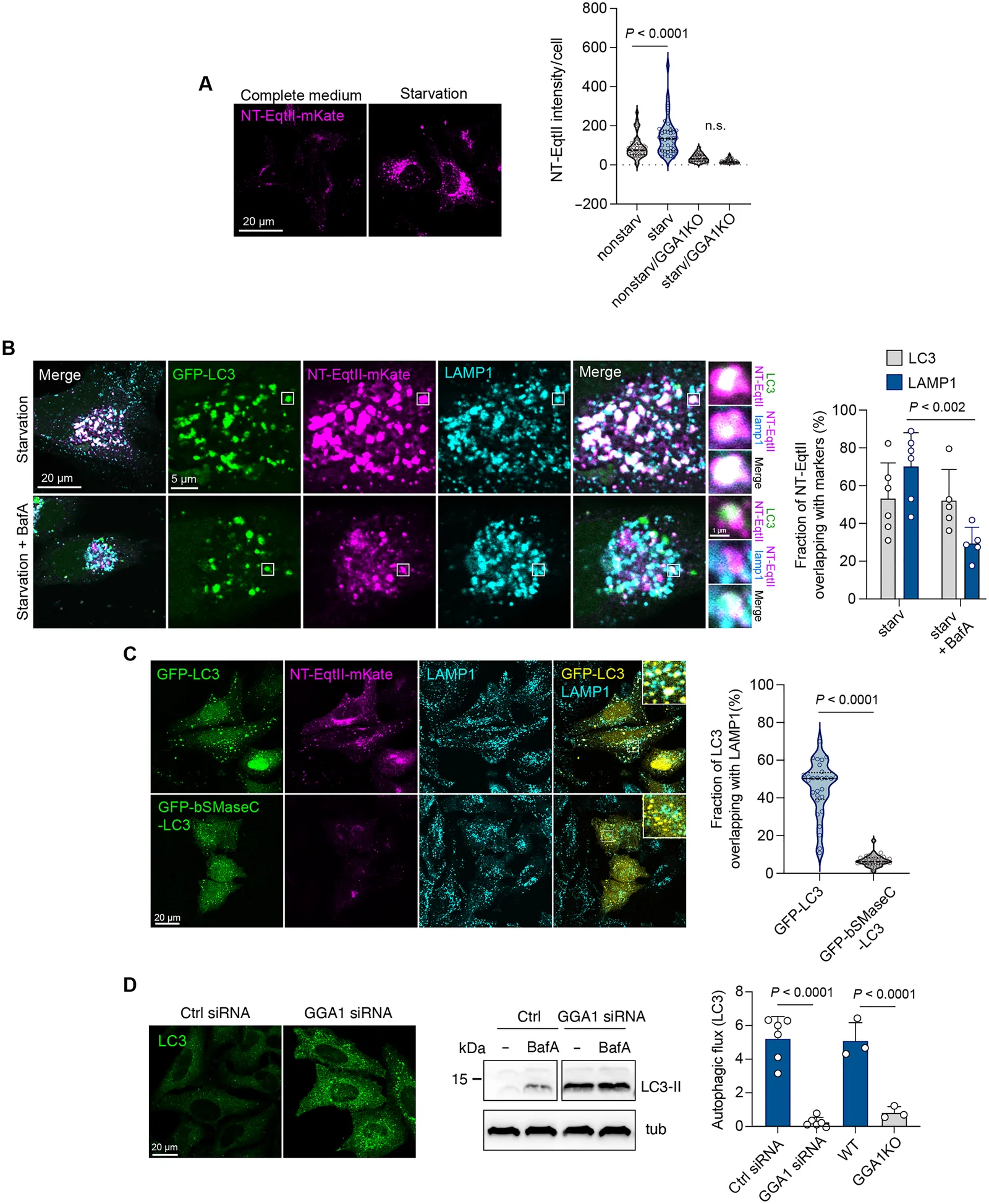
SM exposure to the cytoplasmic leaflet regulates autophagosome-lysosome fusion. (**A**) WT cells were transfected with NT-EqtII-mKate and incubated either for 24 hours in complete medium or for 22 hours in complete medium, followed by 2 hours in Earle’s balanced salt solution (EBSS) to induce starvation. Cells were then fixed and imaged by confocal microscopy. GGA1KO HeLa cells were similarly transfected and treated. NT-EqtII-mKate fluorescence per cell was quantified. *n* = 45 (nonstarved WT), 41 (starved WT), 35 (nonstarved GGA1KO), and 31 (starved GGA1KO). starv, starved. (**B**) WT HeLa cells were cotransfected with GFP-LC3 and NT-EqtII-mKate, incubated for 22 hours in complete medium, and then treated with EBSS for 2 hours with or without 200 nM BafA. Cells were fixed, permeabilized, and immunolabeled with anti-LAMP1 antibody. NT-EqtII-mKate fluorescence overlapping with LC3 or LAMP1 was quantified. Mean ± SD. *n* = 6 (starved LC3), 6 (starved LAMP1), 5 (starved + BafA LC3), and 5 (starved + BafA LAMP1). (**C**) WT cells were cotransfected with GFP-LC3 or GFP-bSMaseC-LC3 and NT-EqtII-mKate. After 24 hours, cells were fixed, permeabilized, and labeled with anti-LAMP1 antibody. The fraction of GFP-LC3 signal overlapping with LAMP1 was quantified. *n* = 28 (GFP-LC3) and 27 (GFP-bSMaseC-LC3). (**D**) Left: WT HeLa cells were treated with either control or GGA1 siRNA for 72 hours, fixed, permeabilized, and labeled with anti-LC3 antibody. Confocal images were acquired. Center: Immunoblot of LC3 from control and GGA1 siRNA–treated cells with or without 2 hours of treatment with 200 nM BafA. LC3-II (lipidated form) and tubulin (loading control) are shown. Right: Autophagic flux was calculated as the difference in LC3-II levels between BafA-treated and untreated cells. *n* = 6 (siRNA conditions) and 3 (WT and GGA1KO). tub, tubulin. Statistical analyses: two-tailed Student’s *t* test [(A) to (D)], pooled at least from two independent experiments.

To further define the role of SM in autophagy, we induced starvation to promote autophagosome formation. Starvation significantly increased NT-EqtII-mKate labeling in WT cells (Fig. 6A) but not in GGA1KO cells, indicating that SM exposure is regulated by GGA1 and is enhanced during nutrient stress.

Upon starvation, NT-EqtII-mKate colocalized with LC3 and LAMP1, indicating transport to autophagolysosomes (Fig. 6B). In addition, NT-EqtII colocalized with WIPI2 (WD-repeat protein interacting with phosphoinositides 2), a marker of early phagophores (fig. S7E) (*65*). In contrast, in bafilomycin A (BafA)–treated cells, which block autophagosome-lysosome fusion (*66*), NT-EqtII colocalized with LC3 and WIPI2 but not with LAMP1 (Fig. 6B). These findings suggest that SM is delivered from the TGN to lysosomes via phagophores and autophagosomes.

To test whether cytoplasmic SM is required for autophagosome-lysosome fusion, we expressed bSMaseC-LC3, which degrades SM on autophagosomal membranes. SM degradation was confirmed by loss of NT-EqtII-mKate signal. LC3-LAMP1 colocalization was markedly reduced in bSMaseC-LC3–expressing cells (Fig. 6C), indicating that cytoplasmic SM is critical for fusion.

We next examined the role of SM in TECPR1 recruitment, which mediates autophagosome-lysosome tethering in aggrephagy, a specialized form of autophagy that involves the selective degradation of protein aggregates in cells (*67*), and has recently been shown to bind SM on damaged lysosomes (*68*, *69*). In line with previous findings, GFP-TECPR1 colocalized with NT-EqtII-mKate following LLOMe-induced lysosomal damage (fig. S7F). However, in GGA1-overexpressing cells, GFP-TECPR1 showed minimal labeling, suggesting that either SM exposure is insufficient or local concentrations are below the recruitment threshold for TECPR1 in this context.

These findings reveal that GGA1 supports autophagy through two mechanisms: (i) SM-dependent trafficking of lysosomal hydrolases and (ii) delivery of SM to phagophores and autophagosomes. To functionally assess GGA1’s role in autophagy, we analyzed LC3 puncta formation in WT, GGA1KO, and GGA1 siRNA–treated cells with or without BafA treatment (Fig. 6D). LC3-positive puncta were elevated under both GGA1-deficient conditions, and autophagic flux (difference in LC3 levels ± BafA) was significantly reduced. These results indicate that GGA1 regulates autophagy by mediating SM exposure on cytoplasmic membranes.

## DISCUSSION

In this study, we demonstrate that GGA1, particularly its GAT domain, promotes the transbilayer movement of SM and Chol to the cytoplasmic leaflet of the TGN in HeLa cells. Model membrane experiments showed that this scrambling activity is dependent on the presence of PE and Chol. Depletion of cytoplasmic SM in the TGN impaired the sorting of MPR and cathepsin D. A subset of the exposed SM was further transported to late endosomes/lysosomes via the autophagic pathway. Removal of SM from autophagosomes inhibited their fusion with lysosomes. These results collectively suggest that GGA1 contributes to the organization of intracellular membranes by facilitating SM exposure on the cytoplasmic leaflet and regulating inter-organelle membrane trafficking.

Our previous work identified PMP2 and GGA1 as regulators of SM transbilayer distribution, with PMP2 acting at the PM and GGA1 acting on internal membranes (*14*). GGAs are conserved cytosolic adaptors recruited to the TGN in an Arf-GTP–dependent manner (*15*, *16*). Among the three mammalian isoforms (GGA1 to GGA3), overexpression of any isoform increased the cytoplasmic detection of SM by SBPs (fig. S1, I and J), while GGA1 knockdown or KO reduced this signal (Fig. 1, A and C). In vitro binding experiments have revealed multidomain interactions among GGAs (*33*). Our observations support the role of GGA1 in mediating SM exposure on the cytoplasmic side of the TGN, possibly via complex formation with other GGA isoforms. GGA1 overexpression did not alter total SM levels or probe expression (Fig. 1G and fig. S1C), suggesting that the increased signal is due to redistribution rather than up-regulation. Together, these findings establish GGA1 as a critical factor promoting SM flipping in the TGN. SM is synthesized in the luminal leaflet of the TGN by SMS1 and SMS2 (*10*, *70*, *71*). We propose that GGA1 facilitates SM translocation to the cytoplasmic leaflet, regulating asymmetry upon subsequent delivery to the PM. Consistent with this, GGA1KO cells accumulated SM in the PM outer leaflet, even in the presence of cytoplasmic bSMase (*14*).

The GAT domain alone was sufficient to mediate SM exposure and lipid scrambling in vitro, promoting flipping of both NBD-labeled SM and PC (Fig. 2, D and E). Although membrane tubulation induced by the GAT domain (Fig. 2F) was correlated with scrambling, it was not sufficient by itself, suggesting an additional requirement for specific lipid composition. Notably, Chol was essential for GAT-induced lipid scrambling (Fig. 3, B to F). The known rapid flip-flop of Chol (*72*, *73*) and its ability to support liquid-ordered domains (*74*) may contribute to the GAT-mediated SM redistribution in the TGN. In contrast to our findings, lipid scrambling by G protein–coupled receptors is inhibited by Chol (*75*). These results suggest differing roles for Chol in membrane protein–mediated versus peripheral protein–mediated lipid scrambling.

Although GAT did not bind SM or Chol directly, it interacted with negatively charged phospholipids such as PI4P (fig. S3A). However, PI4P binding was not observed in reconstituted liposomes, likely due to insufficient PI4P density (fig. S3B). Cryo-EM revealed GAT trimers forming around small vesicles (fig. S3, D to H), indicating a structural basis for its membrane remodeling activity. The GAT domain shares structural similarities with the soluble *N*-ethylmaleimide–sensitive factor attachment protein receptors component syntaxin 1a (*76*), which has been implicated in lipid flipping and vesicle docking (*77*). Moreover, the GAT domain adopts a coiled-coil configuration similar to Bin-Amphiphysin-Rvs domain proteins (*78*, *79*), known to stabilize membrane curvature. PMP2 also induces membrane tubulation but via a β barrel structure (*80*), suggesting that multiple structurally distinct proteins mediate SM transbilayer movement through diverse mechanisms.

In cellular contexts, GGA1-mediated SM exposure required PI4P. PI4K inhibition disrupted GGA1 recruitment and function (fig. S4C), indicating that PI4P-rich domains in the TGN (*81*) support GGA1 assembly and activity. A high local concentration of PI4P may promote accumulation of the GAT domain and facilitate trimer formation in cells. The R260A mutation in the GAT domain disrupted PI4P binding and SM exposure (fig. S4A), although the full-length GGA1 retained activity—likely due to compensatory binding via the VHS domain (*34*). Although Arf1 is known to facilitate membrane tubulation (*82*), its overexpression did not alter SM exposure (fig. S4D), suggesting that GGA1 acts independently of Arf1 in lipid flipping.

GGA proteins are critical for MPR transport from the TGN to lysosomes (*15*). In GGA1KO cells, MPR accumulated in the TGN, and cathepsin D was reduced, mirroring defects observed in SMS1/2 DKO cells (Fig. 4, A to C). Expression of a catalytically active GGA1-bSMaseC chimera also blocked MPR exit from the TGN (Fig. 4D), highlighting the requirement for cytoplasmic SM in proper sorting of lysosomal enzymes. SM synthesis is accompanied by the production of diacylglycerol, which activates protein kinase D, a key regulator of transport carrier formation from the TGN (*83*). It has been proposed that SM itself contributes to the shape and function of the Golgi cisternae (*74*, *84*). Our findings suggest that SM is involved in the exit of GGA1 from the TGN by regulating inositol phospholipid metabolism (Fig. 4, E to H, and fig. S6). The production of PI(4,5)P_2_ in the Golgi is activated by Arf1-GTP (*58*), and GTP loading of Arf family proteins is promoted by positive membrane curvature (*85*). Chol-SM nanodomains have been proposed to stabilize tubular membrane structures (*86*). Thus, one possibility is that SM in the cytoplasmic leaflet stabilizes GGA1-induced positive curvature of the TGN membrane, thereby promoting Arf1 activation and subsequent PI(4,5)P_2_ production. PI(4,5)P_2_ is involved in the recruitment of AP-1 (*87*) and also regulates the binding of Arf1 to Arf GAPs (*59*). In our previous genome-wide screen for proteins that alter interbilayer SM distribution at the PM, we identified the Arf GAP ASAP2 (*14*).

We observed SM on the cytoplasmic leaflet of the PM, nuclear envelope, endosomes, and autophagosomes. GGA1 overexpression also increased cytoplasmic Chol labeling by D4H (Fig. 3G), although its distribution was broader, likely due to nonvesicular transport. Exit of SM from the TGN was inhibited at 20°C and by a GGA1 R260A mutant (Fig. 5D and fig. S4B), implicating GGA1 and vesicular trafficking in SM mobilization. While both SM and MPR exit the TGN through similar mechanisms, they are sorted into distinct downstream pathways—SM traffics to LC3-positive vesicles (autophagosomes), whereas MPR does not. Starvation enhanced cytoplasmic SM exposure and its accumulation in late endosomes/lysosomes, suggesting delivery via autophagy (Fig. 6, A and B). Recent studies have shown enrichment of SM in autophagosomes (*88*). Although autophagosome phospholipids are known to derive from the ER via ATG2) and Vps13 (*89*, *90*), our results identify the TGN as a previously unidentified source of SM. Depletion of SM from autophagosomes inhibited their fusion with lysosomes (Fig. 6C), revealing an essential role for SM in autophagosome maturation. Thus, GGA1 contributes to autophagy not only by transporting lysosomal enzymes but also by regulating SM delivery to autophagosomes.

This study reveals a previously unrecognized function of GGA1 in promoting the transbilayer movement of SM from the luminal to the cytoplasmic leaflet of the TGN. This redistribution of SM is critical for multiple membrane trafficking events, including the proper sorting of lysosomal hydrolases and autophagosome-lysosome fusion. These findings highlight the importance of dynamic lipid asymmetry in intracellular membranes and establish GGA1 as a central regulator of SM distribution and membrane organization under physiological conditions.

## MATERIALS AND METHODS

### Cultured cell lines

HeLa (WT) cells (ATCC and CCL-2) GGA1KO HeLa cells (*14*) (RIKEN BioResource Research Center (BRC) cell bank RCB5659) mCat#8 (control) HeLa cells (*19*, *20*) SMS1/2 DKO HeLa cells (*19*, *20*).

Cells were maintained in Dulbecco’s modified Eagle’s medium (DMEM; Gibco, 21885-025) supplemented with 10% fetal bovine serum (Lonza, Switzerland), penicillin (100 U/ml), and streptomycin (100 μg/ml) at 37°C in a humidified atmosphere with 5% CO_2_. Plasmid constructs were transfected into HeLa using jetPRIME reagent (Polyplus-transfection, France) and incubated for 24 hours. siRNAs were transfected using Lipofectamine RNAiMAX (Thermo Fisher Scientific, 13778) and incubated for 72 hours. For mCAT#8 (Ctrl) and SMS1/2 DKO cells, the culture medium was replaced with DMEM supplemented with 5% LPDS 1 day before the experiment to prevent incorporation of serum-derived SM. For Chol depletion, cells were cultured in medium containing 100 μM lovastatin, 10 μM mevalonate, and 5% LPDS for 24 hours. For starvation experiments, cells were incubated with Earle’s balanced salt solution (EBSS; Sartorius, 02-010-1A).

### SM labeling in the cytoplasmic leaflet of intracellular membranes in HeLa cells

HeLa cells were seeded onto coverslips at a density of 6 × 10^4^ cells per well in a 24-well plate and incubated overnight. The following day, cells were transfected with a SM probe (either fluorescent protein–NT-Lys or NT-EqtII–fluorescent protein) alone or cotransfected with equal amounts of plasmids encoding the gene of interest (e.g., GFP as control or GFP-GGA1) for 24 hours. Cells were then fixed with 4% paraformaldehyde (PFA) and mounted on glass slides using ProLong Diamond Antifade Mountant (Thermo Fisher Scientific). Fixed cells were imaged using either an LSM 700 confocal microscope equipped with a Plan-Apochromat 63×/1.4 numerical aperture (NA) oil immersion objective (Zeiss), an FV1000 confocal microscope with a PlanApo 60×/1.4 NA oil immersion objective (Olympus), or a Leica LCS SP8 confocal microscope equipped with a HCX PL APO 63×/1.40 OIL CS2 objective. Live-cell imaging was performed using the FV1000 system. Representative confocal images were acquired from comparable cellular regions and correspond to optical sections containing the strongest probe-associated fluorescence signal. Under each condition, we imaged controls similarly.

### Thin-layer chromatography

HeLa cells were seeded at a density of 5 × 10^5^ cells per well in a six-well plate and incubated overnight. Cells were then transfected with plasmid DNA using jetPRIME. After 24 hours, cells were washed with ice-cold phosphate-buffered saline (PBS) and lysed in 0.9 ml of 0.1% SDS. Lipids were extracted from the lysate according to Bligh and Dyer (*91*) as follows:

A 0.8-ml aliquot of the lysate (corresponding to 100 μg of protein) was mixed with 1 ml of CHCl_3_ and 2 ml of MeOH, followed by vortexing. Subsequently, 1 ml of CHCl_3_ and 1 ml of 0.1 M KCl were added, and the mixture was vortexed again. After centrifugation at ×635*g*, the lower organic phase was collected and dried under a stream of nitrogen gas.

For alkaline methanolysis, dried lipids were treated with 0.4 ml of 0.1 M KOH in MeOH and incubated at 40°C for 1 hour. The reaction was then neutralized by adding 0.4 ml of 0.1 M HCl in water, followed by vortexing. Lipids were extracted as described above.

The extracted lipids were separated by high-performance thin-layer chromatography (HPTLC; Merck, 1.05633.0001, Germany) using a solvent system consisting of CHCl_3_/MeOH/water [65:25:4 (v/v/v)]. Lipids were visualized by spraying with a cupric acetate solution [3% copper acetate and 8% phosphoric acid and water (v/v)], followed by heating at 180°C for 5 min. The plates were imaged using a LAS-4000 mini system.

### Protein purification

The pGEX6P-GAT plasmid was transformed into *Escherichia coli* BL21 (DE3) competent cells, and protein expression was induced with 0.5 mM isopropyl-β-d-thiogalactopyranoside (Sigma-Aldrich) at 37°C for 4 hours. The harvested cell pellet was resuspended in ice-cold PBS containing a protease inhibitor cocktail (Sigma-Aldrich, 539139), lysed by sonication, and centrifuged at ×12,000*g* for 10 min at 4°C to remove cell debris.

The supernatant was incubated with preequilibrated Glutathione Sepharose 4B beads (GE Life Sciences) for 2 hours at 4°C. The resin was washed with PBS, and the glutathione *S*-transferase (GST) tag was cleaved using PreScission protease (GE Life Sciences) by overnight incubation at 4°C. The cleaved protein was eluted with buffer containing 50 mM tris-HCl (pH 7.4), 150 mM NaCl, 1 mM EDTA, and 1 mM dithiothreitol. For GST-GAT, the washed resin was incubated directly with elution buffer [50 mM tris-HCl (pH 8.0) and 10 mM reduced glutathione] without protease cleavage. Purified GAT was stored at −80°C. NHS-Cy3-GAT was prepared by Cy3 Conjugation Kit (Fast)–Lightning-Link (Abcam, ab188287) according to the manufacturer’s protocol.

### Lipid-protein interaction assays

Lipid-protein interaction assays were performed using Membrane Lipid Strip (Echelon Biosciences, P-6002) according to the manufacturer’s instruction. The membrane was blocked with 1% bovine serum albumin (BSA) in tris-buffered saline containing 0.1% Tween 20 (TBST) for 1 hour at room temperature (RT) and then incubated with GST-GAT (1 μg/ml) protein in the same blocking buffer for 1 hour. Bound GAT was detected using anti-GST antibody (Proteintech, 66001-2-Ig; 1:2000), followed by a horseradish peroxidase (HRP)–conjugated anti-mouse secondary antibody (Sigma-Aldrich, GENA931; 1:3000).

### Liposome preparation

LUVs were prepared from 1 mM total lipid mixtures with the following molar compositions: DOPC:NBD-C12-SM (99:1), DOPC:DOPE:PI4P:Chol:NBD-SM (14:70:5:10:1), DOPC:DOPE:PI4P:Chol:NBD-PC (14:70:5:10:1), DOPC:DOPE:PI4P:Chol:NBD-SM (47:37:5:10:1), DOPC:PI4P:Chol:NBD-SM (84:10:5:1), DOPC:DOPE:Chol:NBD-SM (19:70:10:1), DOPC:DOPE:PI4P:Chol:NBD-SM (4:70:5:20:1), and DOPC:DOPE:PI4P:NBD-SM (24:70:5:1).

Lipid mixtures were dried under a rotary evaporator and further desiccated under vacuum for 1 hour to remove residual solvents. The dried lipid films were rehydrated in 10 mM Hepes buffer containing 150 mM NaCl (pH 7.5) and vortexed for 1 hour to form multilamellar vesicles (MLVs). The MLVs were subjected to 3 cycles of freezing-thawing using liquid nitrogen and a 37°C water bath. The suspensions were then extruded through polycarbonate membranes with a 100-nm pore size (800309, Cytiva) at least 20 times using a Mini Extruder (Avanti Polar Lipids).

GUVs were prepared using the polyvinyl alcohol (PVA) gel–assisted swelling method (*92*) from 1 mM total lipid mixtures in CHCl_3_ with the following molar compositions: DOPC:NBD-SM (99:1), DOPC:DOPE:PI4P:Chol:NBD-SM (14:70:5:10:1), DOPC:DOPE:PI4P:NBD-SM (24:70:5:1).

To prepare PVA-coated coverslips, a 5% (w/w) solution of PVA (MW ∼ 145,000; Sigma-Aldrich) was prepared by dissolving PVA in water at 90°C with constant stirring. Approximately 20 μl of the PVA solution was spread onto 18 mm diameter glass coverslips and dried at 50°C for 30 min. Subsequently, 20 μl of the 1 mM lipid solution was evenly spread on the dried PVA film and placed in a vacuum desiccator for 30 min to remove residual solvent.

GUV formation was initiated by hydrating the dried lipid film with 500 μl of swelling buffer [5 mM Hepes, 100 mM NaCl, and 20 mM sucrose (pH 7.4)], followed by incubation for 1 hour at RT. To assess liposome integrity in the presence or absence of GAT, TRITC-dextran (MW: 10,000; Thermo Fisher Scientific, D1817) was added to the GUV suspension at a final concentration of 50 μg/ml.

### Dithionite assay to determine interbilayer distribution of NBD-labeled lipids

The distribution of NBD-labeled lipids between the inner and outer leaflets of LUV membranes was assessed using a dithionite reduction assay, as previously described (*35*). LUVs (final lipid concentration: 50 μM) containing 1 mol % NBD-labeled lipids in PBS were incubated at 37°C for 60 min in the presence or absence of purified GAT protein in a black 96-well plate, and NBD fluorescence was continuously monitored using a fluorescence plate reader. After 60 s of measurement, freshly prepared sodium dithionite was added to a final concentration of 25 mM. NBD fluorescence was recorded for an additional 5 min.

### Collision quenching

Collision quenching by KI was performed as described (*36*) with slight modification. The dried lipid film composed of DOPC:DOPE:PI4P:Chol:NBD-SM (14:70:5:10:1) was rehydrated in 10 mM Hepes buffer (pH 7.5) containing 1 mM EDTA and 0.25 M KCl, and 1 mM LUVs was prepared. A total of 10 μl LUVs was incubated in 190 μl of 10 mM Hepes buffer containing 40 mM Na_2_S_2_O_3_ and 0.25 M KCl or 0.25 M KI in the absence or presence of 1 μM GAT domain for 30 min at RT in a black 96-well plate, followed by fluorescence measurement. For maximal quenching, vesicles in 0.25 M Kl-containing buffer were solubilized by addition of Triton X-100 (final 1%). *F*_0_ denotes the fluorescence intensity measured in the absence of KI, whereas *F*_(KI)_ represents the fluorescence in the presence of KI.

### Calcein leakage assay

Calcein leakage assay was performed as previously described (*37*) with minor modifications. A dried lipid film composed of DOPC:DOPE:PI4P:Chol:Egg-SM:Rho-PE (14:69:5:10:1:1 molar ratio) was rehydrated in 10 mM Hepes buffer (pH 7.5), containing 150 mM NaCl and 50 mM calcein, and LUVs were prepared. Nonencapsulated calcein was removed by gel filtration on a Sephadex G-50 column equilibrated with 10 mM Hepes (pH 7.5) and 150 mM NaCl.

Fractions containing calcein-loaded liposomes were identified on the basis of turbidity and Triton X-100–induced dequenching of calcein fluorescence and were used immediately for leakage assays. Liposomes were diluted to a final lipid concentration of ∼20 μM and incubated with 1 μM GAT for 30 min at RT in a total volume of 200 μl in black 96-well plates. Fluorescence was measured at the assay endpoint, after which Triton X-100 was added to each well (final concentration: 1%) to induce complete liposome lysis and determine maximal fluorescence (*F*_max_).

GAT-induced calcein leakage was calculated relative to the liposome-only baseline and Triton X-100–induced maximal release. Data are presented as protein-induced calcein leakage (%).

### GUV imaging

GUVs (final lipid concentration: 1 mM) were incubated with 1 μM purified GAT protein in a Gene Frame (25-μl volume, 1.0 by 1.0 cm; Thermo Fisher Scientific, AB0576) on a glass slide, covered with a coverslip, and incubated for 30 min at RT. Confocal fluorescence images were acquired using an FV1000 confocal microscope (Olympus) equipped with a PlanApo 60×/1.4 NA oil immersion objective (Olympus) or a Leica LCS SP8 confocal microscope equipped with a HCX PL APO 63×/1.40 OIL CS2 objective.

### Liposome-coflotation assay

GST-GAT protein (1 μM) was incubated with 1 mM MLVs composed of DOPC:DOPE:PI4P:Chol:NBD-SM [14:70:5:10:1 (mol/mol)] in 150 μl of Hepes-buffered saline [HBS: 20 mM Hepes and 100 mM NaCl (pH 7.5)] containing 0.3 M sucrose. The mixture was incubated for 30 min at RT in an ultracentrifuge tube.

Following incubation, 100 μl of 60% sucrose in HBS was gently added and mixed. This was overlaid sequentially with 200 μl of 25% sucrose in HBS and 200 μl of HBS (0% sucrose). Samples were ultracentrifuged at ×259,000*g* for 30 min at 4°C using Optima MAX-TL ultracentrifuge equipped with TLS-55 swinging-bucket rotor (Beckman Coulter). Fractions (100 μl each) were collected from the top.

The lipid-containing fractions were identified by NBD fluorescence using a fluorescence spectrometer. Collected fractions were analyzed by SDS–polyacrylamide gel electrophoresis (SDS-PAGE), followed by Western blotting.

### Western blotting

Cells were washed with ice-cold PBS and lysed in lysis buffer containing 20 mM Hepes (pH 7.4), 1% Triton X-100, 150 mM NaCl, 5 mM EDTA, and protease inhibitor cocktail (Sigma-Aldrich, 539131). Equal amounts of protein lysates were resolved by SDS-PAGE and transferred onto polyvinylidene difluoride membranes.

Membranes were blocked with 1% (w/v) skim milk in TBST for 30 min at RT and incubated with appropriate primary antibodies at 4°C overnight. After washing with TBST, membranes were incubated with HRP-conjugated secondary antibodies for 1 hour at RT.

Signals were detected using the ECL Prime Western Blotting Detection Reagent (Sigma-Aldrich, GERPN2232) and visualized with the LAS 4000 mini imaging system (GE Healthcare). Band intensities were quantified using the ImageJ software (National Institutes of Health).

### Immunofluorescence microscopy

Cells were grown on coverslips and fixed with 4% PFA for 15 min at RT. After fixation, cells were permeabilized with digitonin (50 μg/ml; Sigma-Aldrich, D141) or 1% Triton X-100 for 10 min and blocked with 1% BSA in PBS for 30 min at RT.

Cells were incubated with primary antibodies for 1 hour at RT, washed with PBS, and then incubated with appropriate secondary antibodies for 1 hour at RT. After rinsing in distilled water, coverslips were mounted on glass slides using ProLong Diamond Antifade Mountant (Thermo Fisher Scientific, P36971).

PI4P and PI(4,5)P_2_ were stained using specific monoclonal antibodies following a modified Golgi staining method (*60*), with PBS used as the buffer and 1% BSA for blocking. Briefly, cells were fixed with 2% PFA for 15 min at RT, permeabilized with 20 μM digitonin for 5 min, and then blocked with 1% BSA in PBS. Cells were subsequently incubated with primary antibodies against PI4P or PI(4,5)P_2_, followed by appropriate secondary antibodies. For PI4P labeling using GFP–PH-OSBP, cells were transfected for 24 hours and then subjected to immunofluorescence staining.

For intracellular Chol staining, cells grown on glass coverslips were fixed with 4% PFA and permeabilized by immersion in a liquid nitrogen bath for 30 s, as previously described (*93*). After thawing, cells were incubated with D4H solution (10 μg/ml in PBS containing 1% BSA) at RT for 1 hour. Following PBS washes, cells were postfixed with 4% PFA for 15 min and mounted.

Cells were imaged using a confocal laser microscope LSM 700 (Zeiss) equipped with a Plan-Apochromat 63×/1.4 NA oil immersion objective, an FV1000 confocal microscope (Olympus) equipped with a PlanApo 60×/1.4 NA oil immersion objective (Olympus) or a Leica LCS SP8 confocal microscope equipped with a HCX PL APO 63×/1.40 OIL CS2 objective.

### Transport of VSV-G

EGFP-tagged VSV-G ts045 (*51*, *52*) was transfected into WT and GGA1KO HeLa cells and expressed for 24 hours at 37°C. To accumulate VSV-G in the TGN, cells were shifted to 20°C for 2 hours and then returned to 37°C for 30 min to allow transport to the PM. Cells were then fixed, and EGFP-VSVG transported to the cell surface was stained with anti-GFP antibody without permeabilization.

### RUSH assay

Str-KDEL-SS-SBP-MMP11-EGFP was transfected into WT and GGA1KO cells for 24 hours. Synchronized release of MMP11-EGFP from the ER was induced by the addition of 40 mM biotin (*53*), and cargo transport from the ER through the Golgi apparatus was monitored by live-cell imaging at 5-min intervals for a total duration of 100 min.

### Isolation of autophagic vesicles and flow cytometry analysis

Autophagic vesicles were isolated on the basis of the protocol described by Schmitt *et al.* (*88*) with slight modifications. HeLa cells at 70% confluency in T75 flasks were transfected with NT-EqtII-mKate. After 24 hours, cells were treated with 200 nM BafA for 2 hours, washed with PBS, harvested by trypsinization, and centrifuged at ×306*g* for 5 min. The cell pellet was resuspended in 0.5 ml of PBS supplemented with EDTA-free protease inhibitor cocktail and disrupted by sonication (Tomy, UR-21P) for 3 × 2 s at 60% amplitude.

The lysate was centrifuged at ×3000*g* for 10 min at 4°C to remove cell debris. The resulting supernatant was further centrifuged at 55,000 rpm (×136,000*g*) for 70 min at 4°C using Optima MAX-TL ultracentrifuge equipped with TLA-55 rotor (Beckman Coulter). The resulting pellet was resuspended in 0.5 ml of PBS and incubated with 2.5 μl of anti-LC3 antibody for 30 min at RT. Subsequently, 2.5 μl of anti–Alexa Fluor 488 (AF488)–conjugated secondary antibody was added and incubated for another 30 min.

The mixture was again centrifuged at 55,000 rpm (×136,000*g*) for 70 min at 4°C as described above. The resulting pellet was resuspended in PBS, and the fluorescence signals of NT-EqtII-mKate and AF488 were analyzed by flow cytometry using a FACSymphony A1 (BD Biosciences).

### Image quantification

Image quantification was performed using Fiji (ImageJ). Background-subtracted images were analyzed to measure mean fluorescence intensity or assessed for colocalization using Mander’s overlap coefficient.

### Cryo–electron microscopy

LUVs at a concentration of 1 to 2 mM were incubated with 25 to 50 μM purified GAT protein for 30 min at RT. For sample preparation, 3 μl of either 50 μM purified GAT alone or GAT preincubated with LUVs was applied to glow-discharged (GloQube Plus, Quorum, 73712) Quantifoil R1.2/1.3 holey carbon 200-mesh copper grids (Quantifoil, 4220C-CF) or Quantifoil UltrAuFoil R2/2 holey gold 300-mesh gold grids (Quantifoil, 4977G-FA). Grids were blotted under 100% humidity at 4°C and vitrified by plunge freezing into liquid ethane using a Vitrobot Mark IV (Thermo Fisher Scientific).

Micrographs were acquired using a Glacios transmission microscope (Thermo Fisher Scientific) operated at 200 kV and equipped with a Falcon 3 EC direct electron detector (Thermo Fisher Scientific). Data collection was performed using EPU software (Thermo Fisher Scientific) in counting mode at a pixel size of 0.95 or 1.6 Å. Each micrograph was dose fractioned into 38 frames, with a total electron dose of 40 e^−^/Å^2^.

All image processing steps were carried out using cryoSPARC v4 (*94*). Micrographs were motion corrected and dose weighted before further analysis. The contrast transfer function was estimated for each micrograph. A subset of micrographs was used for manual particle picking, followed by 2D classification to generate reference class averages. These 2D class averages were then used for template-based particle picking across the entire dataset. Particles selected through this procedure were subjected to two to four rounds of 2D classification to remove false positives and poorly aligned particles.

### Mass photometry

Mass photometry measurements were performed at RT using a Two-Wavelength Optical Mass Photometer (Refeyn). Six-well sample cassettes (Sample Well Cassettes, Refeyn) were placed at the center of MassGlass UC microscope slides (Refeyn). For each measurement, 18 μl of buffer was added to a well to enable autofocus. Following the focusing step, 2 μl of purified GAT protein was added to the well, resulting in a final concentration of 40 nM. Measurements were acquired immediately after mixing.

### Protein modeling

Structural models of the GAT domain were generated using AlphaFold 3 (*45*) via the AlphaFold server (https://alphafoldserver.com) with default parameters. Both monomeric and trimeric forms of the GAT domain were modeled. To simulate a 2D projection, the trimer model was converted into an electron density map at a resolution of 6 Å using the molmap command in ChimeraX (*95*).

### APEX analysis

APEX EM analysis was undertaken as described previously (*96*). Briefly, cells were transfected with APEX2-GBP (*96*) for detection of GFP-tagged proteins and EGFP-P2A-AEX2-csAPEX for detection of Cherry-tagged proteins. Transfection efficiency and fluorescence signal were checked using live cell imaging on an EVOS FL epifluorescence microscope (Thermo Fisher Scientific).

Cells were then fixed with 2.5% glutaraldehyde, washed repeatedly with PBS, and incubated with diaminobenzidine in the presence of hydrogen peroxide. Samples were postfixed with 1% osmium tetroxide and then serially dehydrated in ethanol. Cells were serially infiltrated with LX112 resin and polymerized overnight at 60°C. Approximately 70-nm sections were obtained using an ultramicrotome (Leica EM UC6, Leica Microsystem) and images acquired using a JEOL 1011 transmission electron microscope at 80 kV.

## QUANTIFICATION AND STATISTICAL ANALYSIS

All data were pooled from at least two independent experiments. Statistical significance was assessed using a two-tailed Student’s *t* test for comparison between two groups or one-way analysis of variance (ANOVA), followed by Tukey’s post hoc test for multiple comparisons. The number of biological replicates (*n*) used for statistical analysis is indicated in figure legends. *P* < 0.05 was considered statistically significant. Data are presented as the median or mean ± SD, as specified.

## Supplementary Materials

### The PDF file includes:

Figs. S1 to S7 Table S1 Legends for movies S1 to S9 References
