# Supplementary material for: Sorting of lysosomal enzyme and autophagy are regulated by the GGA1-induced TGN lipid scrambling
Source: Sci Adv. 2026 Aug 28;12(35):eaec4519. doi: 10.1126/sciadv.aec4519 (PMC13524056; doi:10.1126/sciadv.aec4519)
Supplement: Supplementary file 1 [file sciadv.aec4519_sm.pdf]

Science Advances
AAAS

Supplementary Materials for

Sorting of lysosomal enzyme and autophagy are regulated by the GGA1-induced TGN lipid scrambling

Kohta Takahashi *et al.*

Corresponding author: Kohta Takahashi, kohta.takahashi.x@niigata-u.ac.jp;
Toshihide Kobayashi, toshihide.kobayashi@unistra.fr

Sci. Adv. **12**, eaec4519 (2026)
DOI: 10.1126/sciadv.aec4519

The PDF file includes:

Figs. S1 to S7
Table S1
Legends for movies S1 to S9
References

Other Supplementary Material for this manuscript includes the following:

Movies S1 to S9

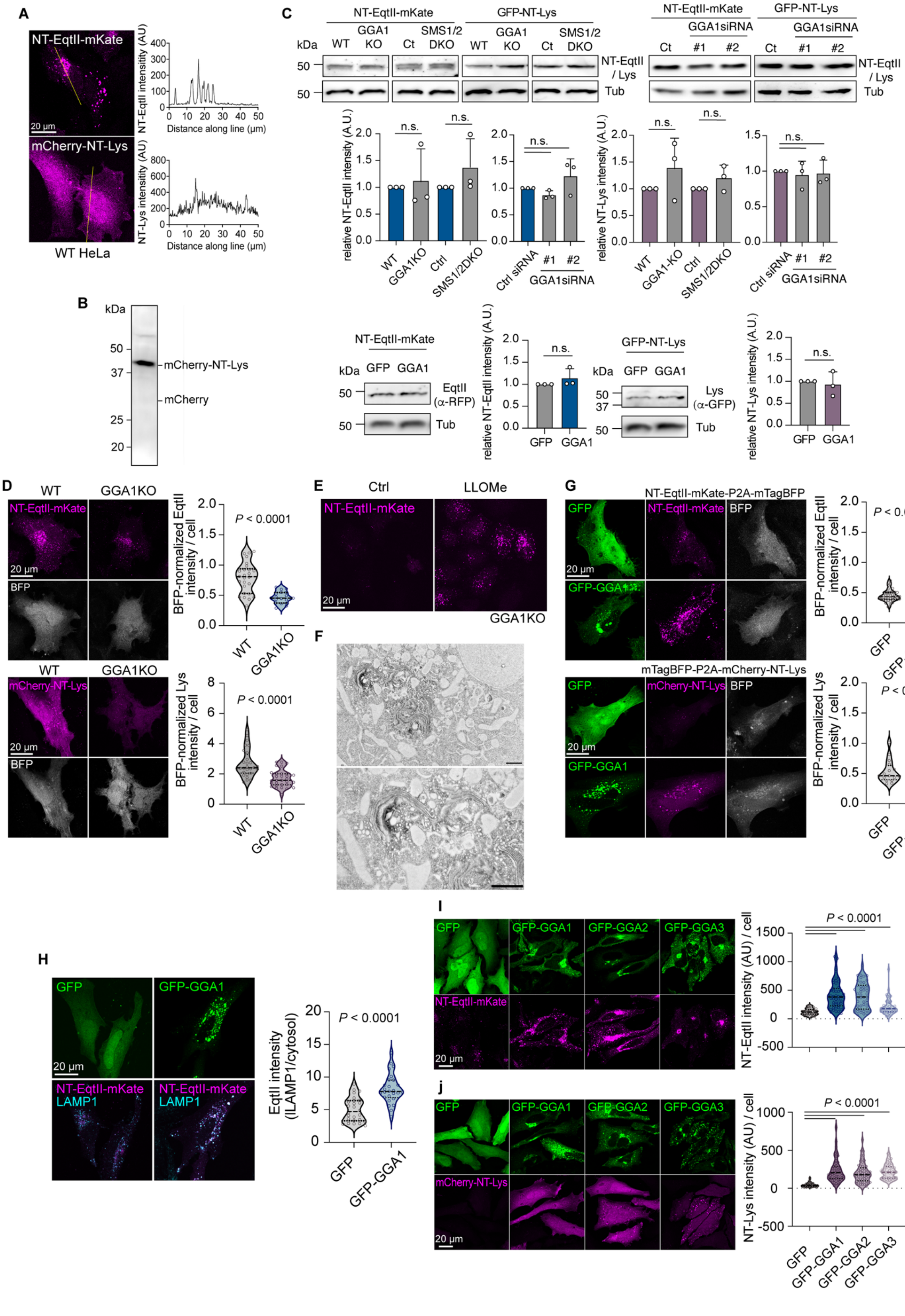
A
NT-EqtII-mKate
20 µm
NT-EqtII intensity (AU)
Distance along line (µm)
mCherry-NT-Lys
NT-Lys intensity (AU)
Distance along line (µm)
WT HeLa
B
kDa
50
37
25
20
mCherry-NT-Lys
mCherry
C
NT-EqtII-mKate
GFP-NT-Lys
kDa
WT
GGA1 KO
Ct
SMS1/2 DKO
NT-EqtII / Lys
Tub
GGA1siRNA
Ct
#1
#2
relative NT-EqtII intensity (A.U.)
n.s.
WT
GGA1KO
Ctrl
SMS1/2DKO
Ctrl siRNA
GGA1siRNA
relative NT-Lys intensity (A.U.)
GGA1-KO
GFP
GGA1
EqtII (α-RFP)
Lys (α-GFP)
D
WT
GGA1KO
NT-EqtII-mKate
BFP
20 µm
BFP-normalized EqtII intensity / cell
P < 0.0001
mCherry-NT-Lys
BFP-normalized Lys intensity / cell
E
Ctrl
LLOMe
NT-EqtII-mKate
20 µm
GGA1KO
F
G
NT-EqtII-mKate-P2A-mTagBFP
GFP
GFP-GGA1
BFP
BFP-normalized EqtII intensity / cell
P < 0.0001
mTagBFP-P2A-mCherry-NT-Lys
BFP-normalized Lys intensity / cell
P < 0.002
H
GFP
GFP-GGA1
20 µm
NT-EqtII-mKate
LAMP1
EqtII intensity (lLAMP1/cytosol)
P < 0.0001
I
GFP
GFP-GGA1
GFP-GGA2
GFP-GGA3
NT-EqtII-mKate
20 µm
NT-EqtII intensity (AU) / cell
P < 0.0001
j
mCherry-NT-Lys
NT-Lys intensity (AU) / cell
P < 0.0001

Fig. S1. GGA proteins mediate the exposure of sphingomyelin on the cytosolic leaflet. (**A**) Representative confocal images of HeLa cells expressing NT-EqtII-mKate or mCherry-NT-Lys to visualize sphingomyelin (SM) exposed on cytoplasmic leaflet of the intracellular membranes. NT-EqtII-mKate predominantly labeled discrete intracellular puncta, whereas mCherry-NT-Lys exhibited broader intracellular distribution with local enrichment. Fluorescence intensity profile measured along the indicated line (yellow) are shown on the right. Scale bar, 20 µm. (**B**) Immunoblot analysis of mCherry-NT-Lys expressed in HeLa cells. Full-length mCherry-NT-Lys was detected with negligible free mCherry, indicating that nuclear labeling was unlikely to arise from cleavage of the probe. (**C**) Immunoblot analysis of NT-EqtII-mKate and GFP-NT-Lys expression under conditions used for fluorescence imaging experiments. Top, wild-type, GGA1 KO, control, and SMS1/2 DKO HeLa cells, or cells treated with control or GGA1 siRNA (#1 and #2), were transfected with NT-EqtII-mKate or GFP-NT-Lys and analyzed by immunoblotting using anti-TagRFP (NT-EqtII-mKate) or anti-GFP (GFP-NT-Lys) antibodies. Tubulin (Tub) served as a loading control. Bottom, wild-type HeLa cells were co-transfected with GFP or GFP-GGA1 together with NT-EqtII-mKate or GFP-NT-Lys and analyzed similarly. Relative band intensities were normalized to corresponding controls and quantified (n = 3 independent experiments, mean ± SD; paired Student's *t*-test). No significant changes in probe expression levels were observed under these conditions. (**D**) NT-EqtII-mKate-P2A-mTagBFP2 (Top) or mTagBFP2-P2A-mCherry-NT-Lys (Bottom) were transfected into WT and GGA1 KO cells. Fluorescence ratios of mKate/BFP and mCherry/BFP were quantified. (Top: n = 26 and 18, Bottom: n = 28 and 30). (**E**) GGA1 KO cells were transfected with NT-EqtII-mKate and treated with 250 µM LLOMe for the final 30 min of a 24 h incubation. Confocal images were acquired. (**F**) Wild-type HeLa cells were co-transfected with mCherry-GGA1 and EGFP-P2A-APEX2-ChBP, followed by APEX staining. Specific labeling was observed at the Golgi complex as shown at low magnification in the upper panel and at higher magnification (of the same cellular region) in the lower panel. Scale bars: 1000 nm. (**G**) HeLa cells were transfected with NT-EqtII-mKate-P2A-mTagBFP2 (Top) or mTagBFP2-P2A-mCherry-NT-Lys (Bottom) together with either GFP or GFP-GGA1. Fluorescence ratios of mKate/BFP and mCherry/BFP were quantified. (Top: n = 38 and 41, Bottom: n = 16 and 18). (**H**) WT HeLa cells were co-transfected with NT-EqtII-mKate and either GFP or GFP-GGA1. After 24 h, cells were fixed, permeabilized, and labeled with anti-LAMP1 antibody. NT-EqtII-mKate fluorescence intensity associated with LAMP1-positive compartments relative to cytosolic fluorescence was quantified. n = 26 (GFP), 26 (GFP-GGA1). (**I**) WT HeLa cells were co-transfected with NT-EqtII-mKate and either GFP, GFP-GGA1, GFP-GGA2, or GFP-GGA3. NT-EqtII-mKate fluorescence was quantified (n = 82, 98, 67, and 65, respectively). (**J**) WT cells were co-transfected with mCherry-NT-Lys and the same GFP-tagged constructs as in (**I**). Fluorescence intensity of mCherry-NT-Lys was quantified (n = 50, 46, 54, 45, respectively). Statistical analyses: paired (**C**) and two-tailed (**D**, **G**, **H**, **I**, **J**) Student's t test. Data were pooled from at least two independent experiments.

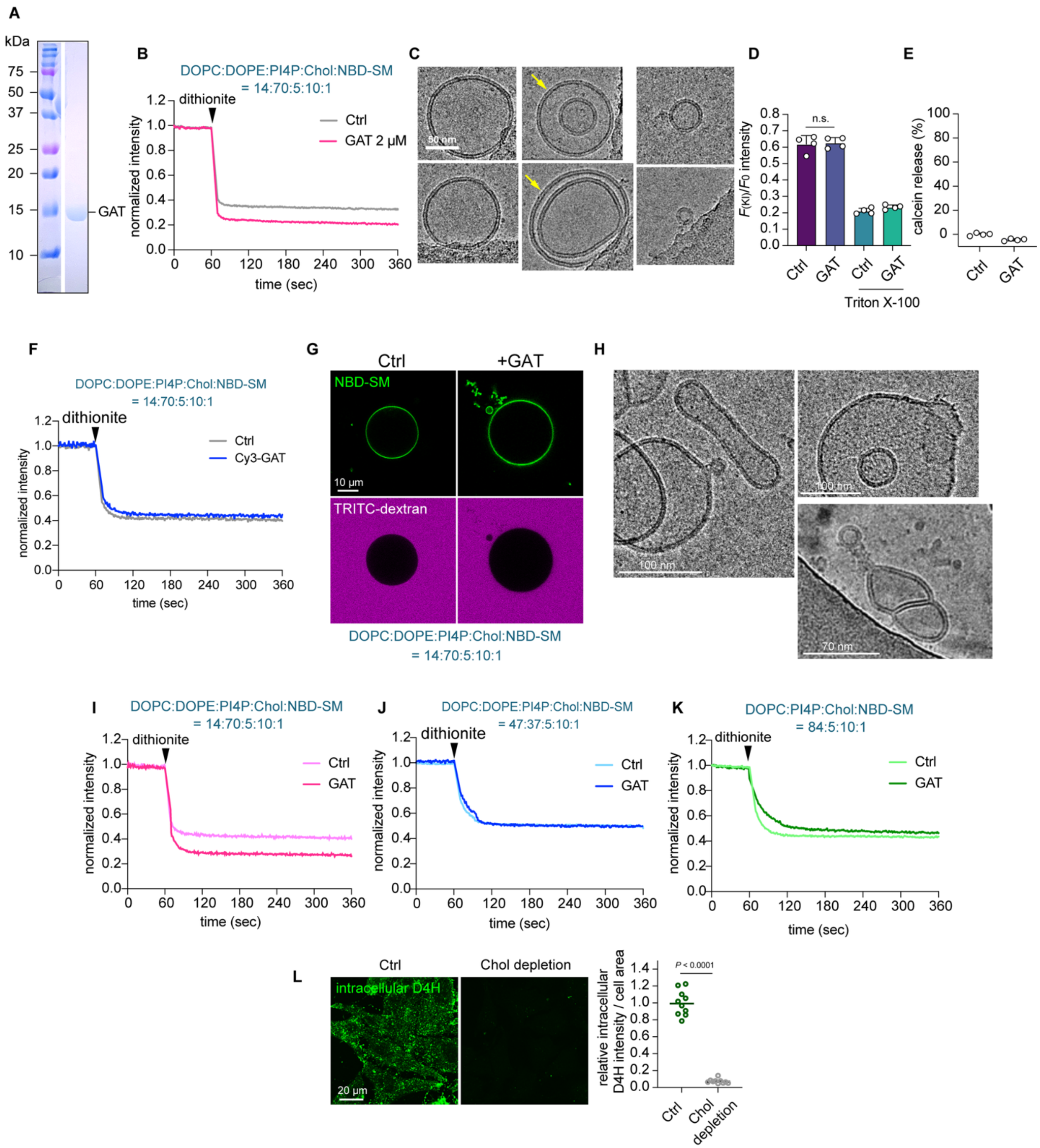

Fig. S2. The GAT domain of GGA1 promotes lipid scrambling. (**A**) SDS-PAGE of purified GGA1 GAT domain. (**B**) LUVs composed of DOPC:DOPE:PI4P:Chol:NBD-SM (14:70:5:10:1) were treated with or without 2 µM GAT. Dithionite treatment and fluorescence analysis were performed as described in Fig. 2C-E. *n* = 3-4. (**C**) Cryo-EM micrographs of LUVs. Arrows indicate double lamellar liposomes. (**D**) Collision quenching assay. LUVs composed of DOPC:DOPE:PI4P:Chol:NBD-SM (14:70:5:10:1) were incubated in the absence or presence of the 1 µM GAT domain -/+ 0.25 M KI as described in **MATERIALS AND METHODS**. For maximal quenching, vesicles were solubilized by addition of 1% Triton X-100. F_0 denotes the fluorescence intensity measured in the absence of KI, whereas $F_{(KI)}$ represents the fluorescence in the presence of KI. Mean ± SD. *n* = 4. (**E**) Calcein leakage assay. Calcein-loaded LUVs composed of

DOPC:DOPE:PI4P:Chol:Egg-SM:Rho-PE (14:69:5:10:1:1) were incubated with 1 µM GAT at a final lipid concentration of approx. 20 µM. Calcein fluorescence was measured at the assay endpoint, followed by addition of Triton X-100 to induce complete liposome lysis and determine maximal fluorescence (F_{max}). Calcein release was calculated relative to liposome-only baseline and Triton X-100-induced maximal release. Data are presented as calcein release (%). Mean ± SD**.** *n* = 4. (**F**) LUVs composed of DOPC:DOPE:PI4P:Chol:NBD-SM (14:70:5:10:1) were treated with or without NHS-Cy3-modified GAT. Dithionite treatment and fluorescence analysis were performed as described above. *n* = 3-4. (**G**) GUVs composed of DOPC:DOPE:PI4P:Chol:NBD-SM (14:70:5:10:1), were incubated ± 1 µM GAT protein for 30 min at RT in the presence of 50 µg/mL TRITC-dextran. Fluorescence images were acquired by confocal microscopy. Tubules and small vesicles were associated to GAT-treated GUVs. (**H**) Cryo-EM micrographs of LUVs incubated with GAT protein, as described in **MATERIALS AND METHODS**. (**I**-**K**) LUVs with various lipid compositions-(**I**) DOPC:DOPE:PI4P:Chol:NBD-SM (14:70:5:10:1), (**J**) DOPC:DOPE:PI4P:Chol:NBD-SM (47:37:5:10:1), (**K**) DOPC:DOPE:Chol:NBD-SM (19:70:10:1)-were treated with or without 1 µM GAT. Dithionite treatment and fluorescence analysis were performed as described above. *n* = 3-4. (**L**) WT HeLa cells were Chol-depleted using 100 µM lovastatin and 10 µM mevalonate in 5% LPDS for 24 h. Cells were then fixed, permeabilized and labeled with sfGFP-D4H. Fluorescence images were acquired by confocal microscopy. sfGFP-D4H fluorescence per cell was quantified (*n* = 10). Statistical analyses: two-tailed Student's *t* test (**D**, **E**, **G**). Data were pooled from at least two independent experiments.

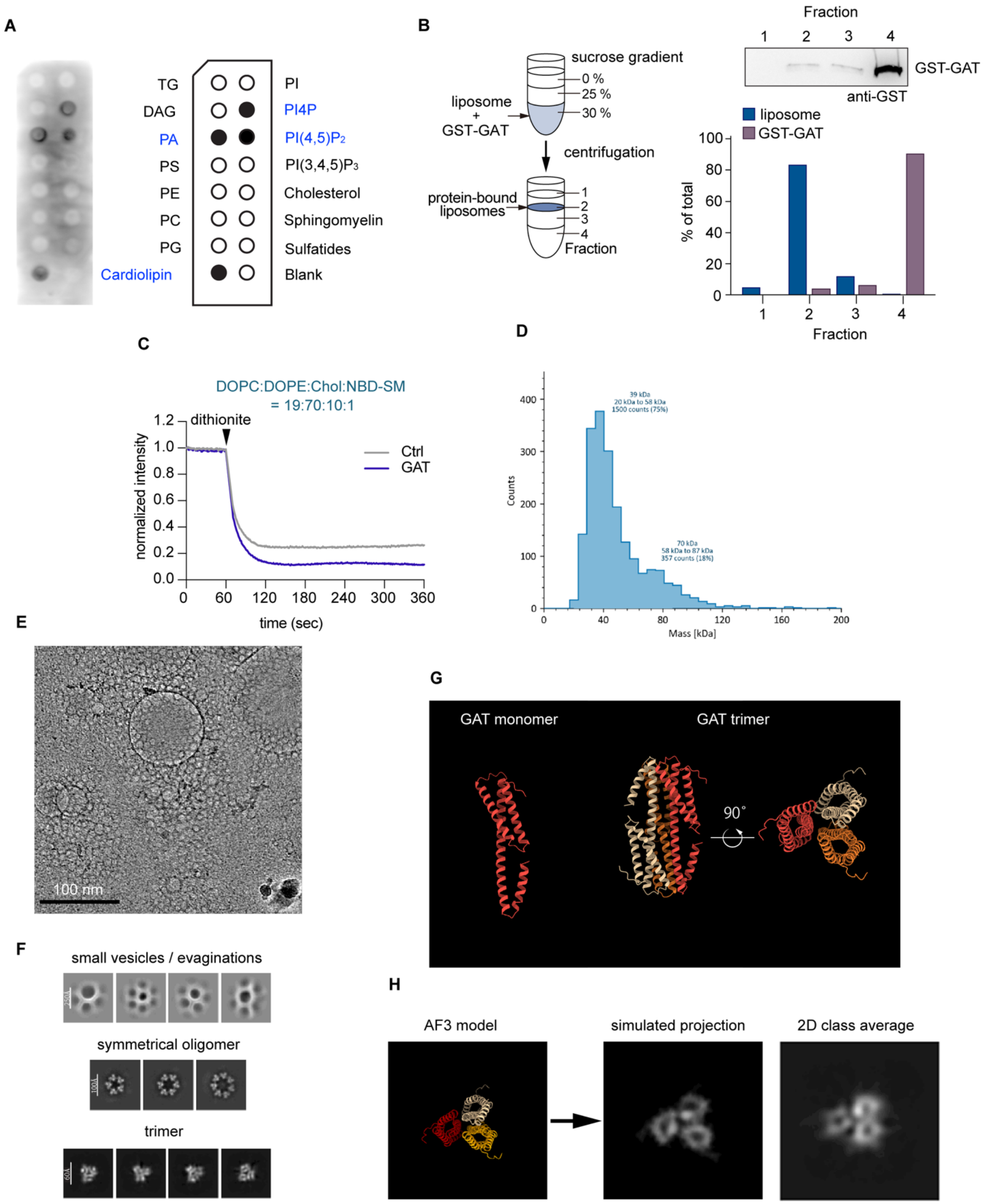

Fig. S3. PI4P is not required for GAT-mediated lipid scrambling in liposomes and the GAT domain forms trimers. (**A**) GST-GAT binding to various lipids was assessed using a Membrane Lipid Strip. Experimental results (left) and schematic summary (right) are shown. The names of the lipids with positive signals are indicated in blue. TG: triacylglycerol, DAG: diacylglycerol, PA: phosphatidic acid, PS: phosphatidylserine, PC: phosphatidylcholine, PE: phosphatidylethanolamine, PG: phosphatidylglycerol,

PI: phosphatidylinositol, $PI(3,4,5)P_3$: phosphatidylinositol 3,4,5-trisphosphate. (**B**) (left) Schematic of liposome co-flotation assay using MLVs composed of DOPC:DOPE:PI4P:Chol:NBD-SM (14:70:5:10:1). Bound protein was recovered in Fraction 2. (upper right) Fractions were analyzed by Western blot with anti-GST antibody. (lower right) NBD fluorescence (dark blue) and GST-GAT band intensity (purple) were quantified. (**C**) LUVs composed of DOPC:DOPE:Chol:NBD-SM (19:70:10:1) were incubated with or without 1 µM GAT protein. NBD fluorescence was monitored during dithionite quenching; values were normalized to pre-quenching levels. Data represent mean values from 3–4 independent experiments. (**D**) Mass distribution histogram from mass photometry landing assays. The average molecular mass of GAT in solution was ~39 kDa, consistent with the expected mass of the trimer. (**E**) Representative cryo-EM micrograph showing 100 nm LUVs incubated with GAT, surrounded by smaller vesicles. (**F**) 2D class averages of the smaller vesicles reveal visible bilayers, symmetrical oligomers, and trimeric GAT assemblies. Scale bar: 250 Å (upper), 100 Å (middle), 60 Å (lower). (**G**) AlphaFold 3 models of monomeric and trimeric GAT domains. (**H**) Comparison of a simulated projection of the AlphaFold 3-modeled GAT trimer with a representative 2D class average from panel **F**.

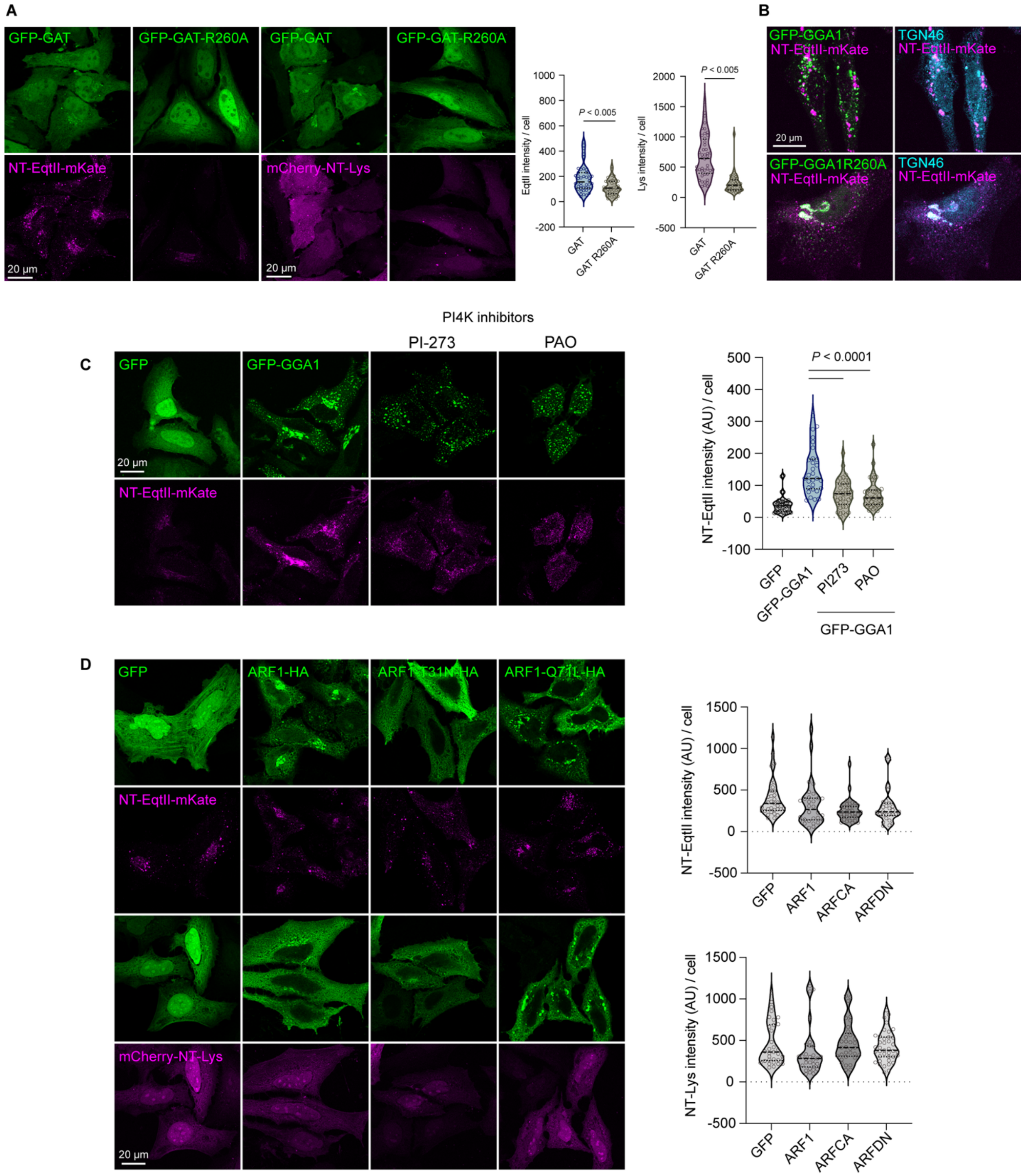

Fig. S4. PI4P, but not Arf1, is required for GGA1-mediated exposure of sphingomyelin. (**A**) WT HeLa cells were transfected with GFP-GAT or GFP-GAT R260A, along with NT-EqtII-mKate or mCherry-NT-Lys. Confocal images were acquired after 24 h. Fluorescence intensity per cell was quantified. *n* = 42 (GAT + NT-EqtII-mKate), 40 (GAT R260A + NT-EqtII-mKate), 44 (GAT + mCherry-NT-Lys), 38 (GAT R260A + mCherry-NT-Lys); pooled from two independent experiments. (**B**) WT HeLa cells were co-transfected with GFP-GGA1 or GFP-GGA1 R260A mutant and NT-EqtII-mKate. After 24 h, cells were fixed, permeabilized, and immunolabeled with anti-TGN46 antibody. (**C**) WT HeLa cells were transfected with GFP or GFP-GGA1 together with NT-EqtII-mKate. During the final 4 h, GFP-GGA1-transfected cells were

treated with 4 µM PI-273 or 1 µM PAO. Cells were then fixed, and confocal images were acquired. NT-EqtII-mKate fluorescence intensity per cell was quantified. *n* = 30 (GFP), 27 (GFP-GGA1), 21 (GFP-GGA1 + PI-273), 17 (GFP-GGA1 + PAO). (**D**) WT HeLa cells were transfected with GFP, GFP-Arf1-HA, constitutively active GFP-Arf1 Q71L-HA, or dominant-negative GFP-Arf1 T31N-HA, together with NT-EqtII-mKate or mCherry-NT-Lys. After 24 h, cells were fixed, permeabilized, and immunolabeled with anti-HA antibody. Fluorescence intensity per cell was quantified. Upper panel (NT-EqtII-mKate): *n* = 33 (GFP), 30 (Arf1-HA), 27 (Q71L), 35 (T31N). Lower panel (mCherry-NT-Lys): *n* = 33 (GFP), 29 (Arf1-HA), 39 (Q71L), 33 (T31N). Statistical analyses: two-tailed Student's *t* test (**A**, **C**). Data were pooled from at least two independent experiments.

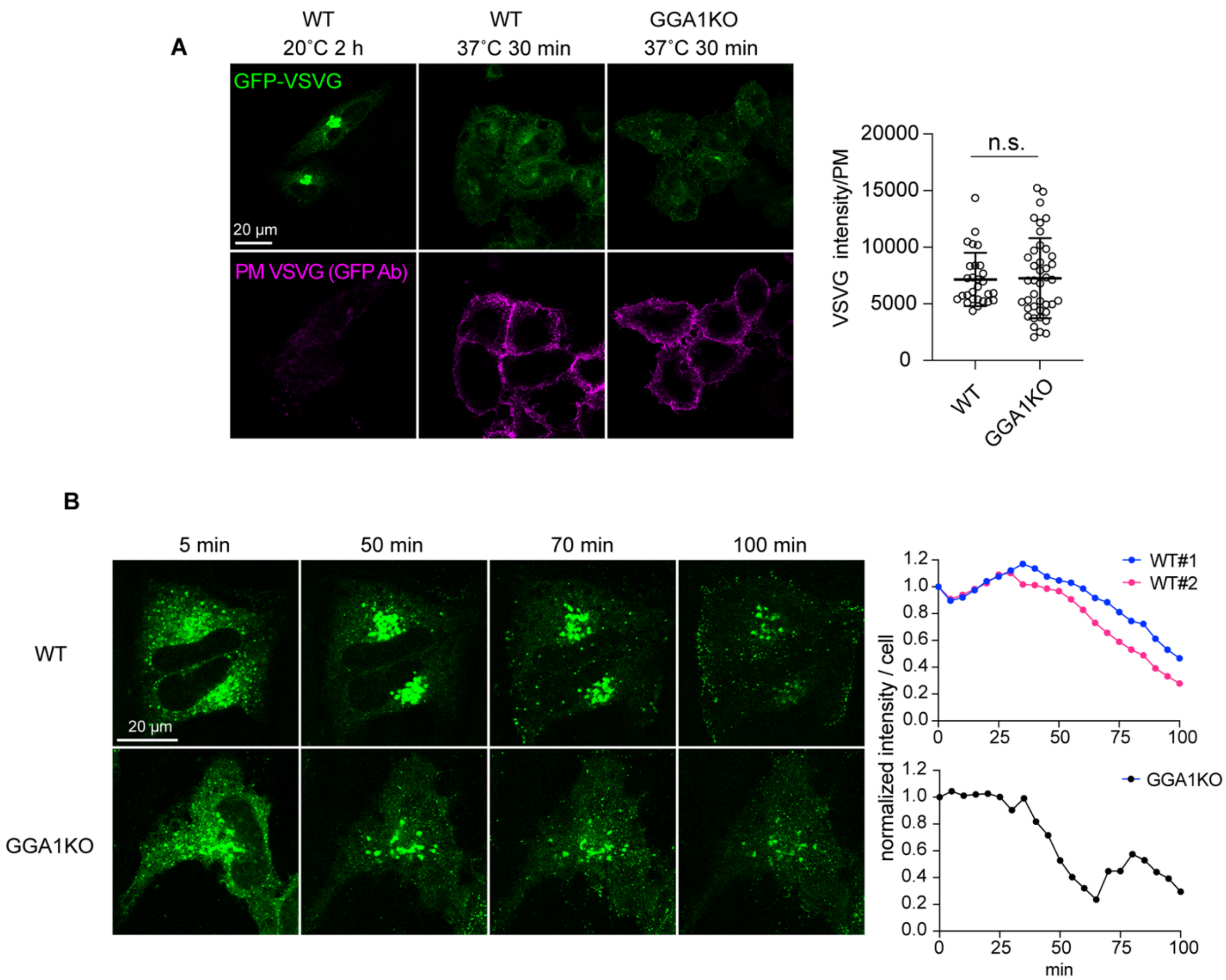

Fig. S5. Effect of SM scrambling on the transport of cell surface protein and protein secretion. (**A**) GFP-VSVG was transfected with WT and GGA1 KO HeLa cells for 24 h at 37 °C. Cells were shifted to 20 °C for 2 h to accumulate VSVG at the TGN followed by 30 min incubation at 37 °C. Cell surface GFP-VSVG was visualized by anti-GFP antibody without permeabilization. PM VSVG fluorescence intensity per cell was quantified. Mean ± SD. n = 28 (WT) and 41 (GGA1KO). (**B**) The RUSH system was used to monitor secretory cargo trafficking. KDEL mediated ER retention of Str-KDEL-SS-SBP-MMP11-EGFP, which was expressed in WT and GGA1KO HeLa cells for 24 h. Cargo release from the ER was triggered by the addition of 40 mM biotin, and secretion of MMP11-EGFP from the ER through the Golgi was followed by live-cell imaging over 100 min. Fluorescence intensity per cell at each timepoint was quantified. Fluorescence at 0 min was set to 1; relative fluorescence was calculated. WT#1 indicates quantification of upper, and WT#2 indicates lower cell from left panel, respectively. Statistical analyses: two-tailed Student's t-test (**A**), pooled from two independent experiments.

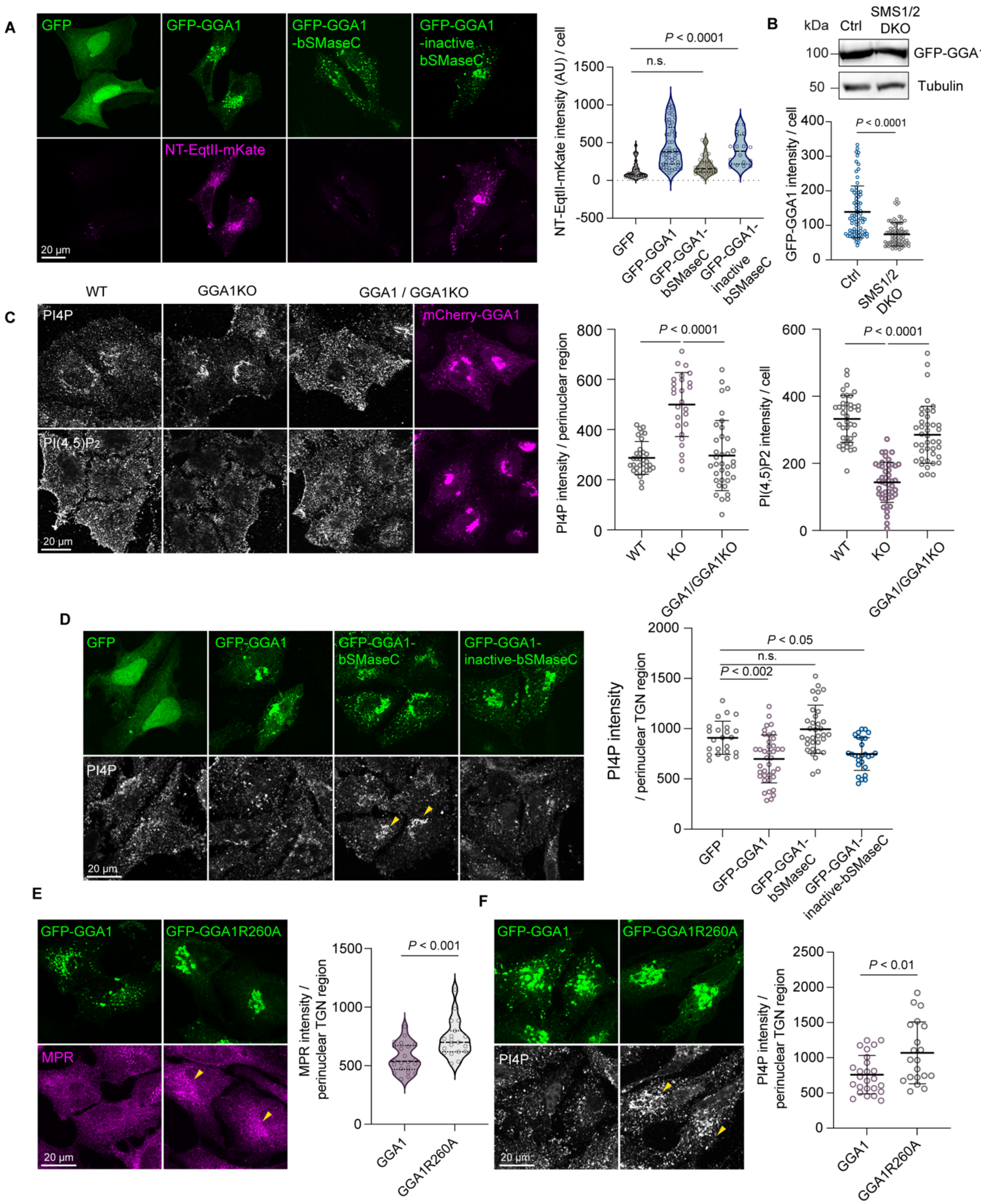

Fig. S6. SM exposure regulates PI4P metabolism. (**A**) WT HeLa cells were transfected with GFP, GFP-GGA1, GFP-GGA1-bSMaseC, or a catalytically inactive mutant (GFP-GGA1-bSMaseC D321A/H322A), along with NT-EqtII-mKate. Confocal images were acquired 24 h post-transfection. NT-EqtII-mKate fluorescence intensity per cell was quantified. *n* = 48 (GFP), 51 (GFP-GGA1), 40 (GFP-GGA1-bSMaseC), 18 (GFP-GGA1-inactive bSMaseC). (**B**) (upper) GFP-GGA1 was expressed in control and SMS1/2 DKO HeLa

cells and analyzed by Western blot. (lower) GFP-GGA1 fluorescence intensity per cell was quantified. Mean ± SD. *n* = 77 (Ctrl), 77 (SMS1/2 DKO). (**C**) (left four panels) PI4P and PI(4,5)P_2 were labeled in wild-type and GGA1 KO cells using specific antibodies. (right four panels) GGA1 KO cells were transfected with mCherry-GGA1 and labeled for PI4P and PI(4,5)P_2. (right) Quantification of PI4P in the perinuclear region and PI(4,5)P_2 per cell. Mean ± SD. *n* = 33 (PI4P, WT), 26 (PI4P, GGA1 KO), 36 (PI4P, rescued); *n* = 39 (PI(4,5)P_2, WT), 46 (GGA1 KO), 41 (rescued). (**D**) WT HeLa cells were transfected with GFP, GFP-GGA1, GFP-GGA1-bSMaseC, or GFP-GGA1-inactive bSMaseC. After 24 h, cells were fixed and labeled for PI4P. PI4P fluorescence intensity in the perinuclear region was quantified. Mean ± SD. *n* = 23 (GFP), 39 (GFP-GGA1), 34 (bSMaseC), 27 (inactive bSMaseC). (**E**) WT HeLa cells were transfected with GFP-GGA1 or GFP-GGA1 R260A. CI-MPR was labeled after 24 h. CI-MPR fluorescence intensity in the perinuclear region was quantified. *n* = 22 (GFP-GGA1), 19 (GFP-GGA1 R260A). (**F**) WT HeLa cells were transfected with GFP-GGA1 or GFP-GGA1 R260A. After 24 h, PI4P was labeled. (right) PI4P fluorescence intensity in the perinuclear region was quantified. Mean ± SD. *n* = 25 (GFP-GGA1), 20 (GFP-GGA1 R260A). Statistical analyses: two-tailed Student's *t* test (**B**, **E**, **F**) and one-way ANOVA with Tukey's post hoc test (**A**, **C**, **D**). Data were pooled from at least two independent experiments.

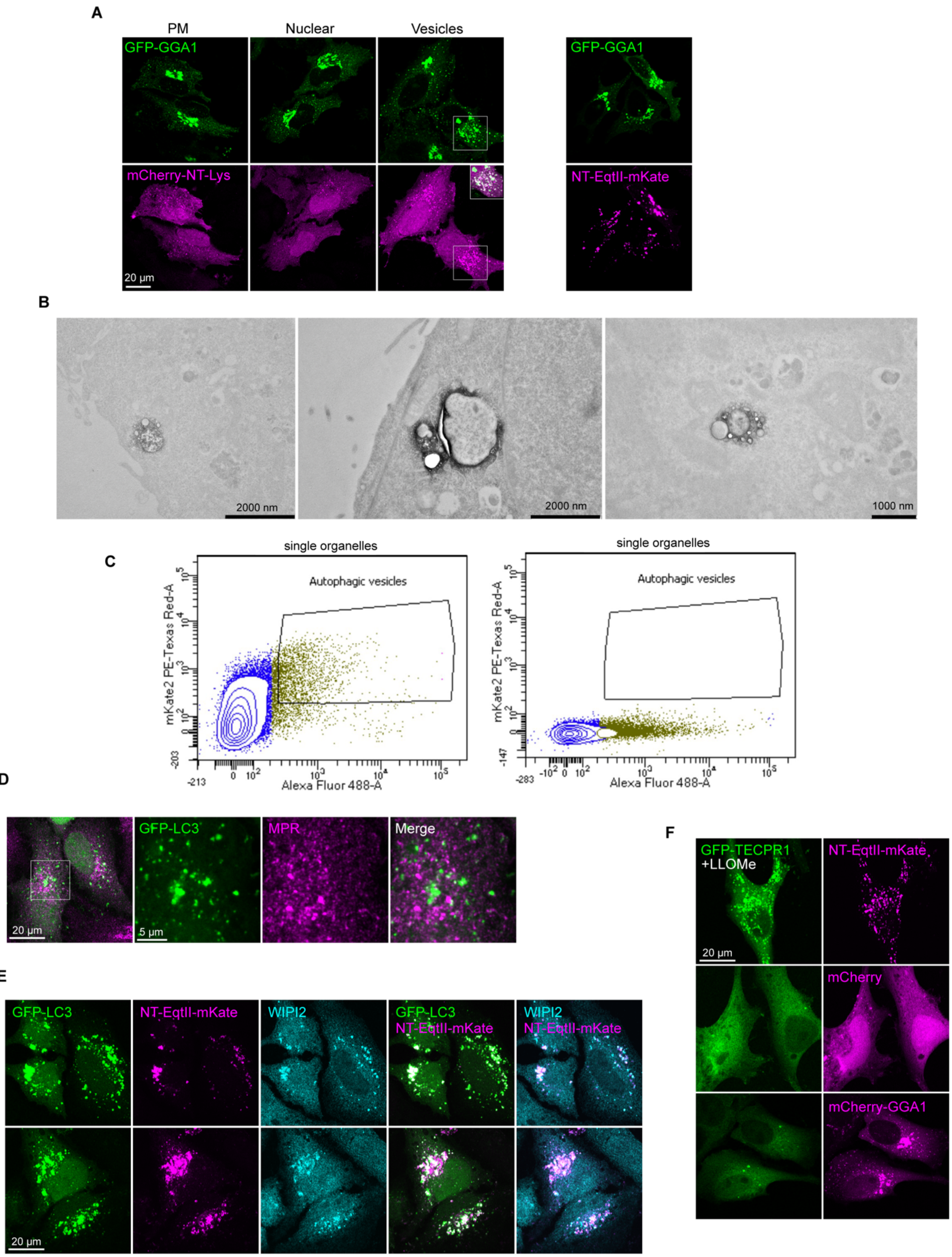

Fig. S7. Exposed SM is transported to phagophores and autophagosomes. (**A**) (left) WT HeLa cells were transfected with GFP-GGA1 and mCherry-NT-Lys. (right) Cells were transfected with GFP-GGA1 and NT-EqtII-mKate to visualize SM localization in intracellular organelles. Confocal images were acquired after

24 h. (**B**) APEX EM detection of SM in cells overexpressing either pEGFP-NT-Lys (left) or NT-EqtII-mClover3 (center and right), co-transfected with mCherry-GGA1, using the APEX2-GBP construct. (**C**) Flow cytometric analysis of autophagic vesicles labeled with NT-EqtII-mKate (y-axis) and LC3 antibody (x-axis), derived from wild-type (left) and GGA1 KO (right) cells. (**D**) WT HeLa cells were transfected with GFP-LC3. After 24 h, cells were fixed, permeabilized, and stained with an anti-CI-MPR antibody. Confocal imaging followed. (**E**) Cells were co-transfected with GFP-LC3 and NT-EqtII-mKate. After 24 h, cells were fixed, permeabilized, and labeled with an anti-WIPI2 antibody. Confocal imaging was performed. (**F**) (top) WT HeLa cells transfected with GFP-TECPR1 and NT-EqtII-mKate were treated with 250 μM LLOMe for the final 30 min. (middle) Cells were transfected with GFP-TECPR1 and mCherry. (bottom) Cells were transfected with GFP-TECPR1 and mCherry-GGA1. All confocal images were acquired 24 h post-transfection.

Table S1. Reagent or resource used in this study.

REAGENT OR RESOURCE	SOURCE	IDENTIFIER
Antibodies		
Rabbit monoclonal anti-GGA1 antibody	Abcam	Cat. # ab170956
Rabbit polyclonal anti-TGN46 antibody	Abcam	Cat. # ab50595
Rabbit polyclonal anti-GFP antibody	Abcam	Cat. # ab290
Rabbit polyclonal anti-CI-MPR antibody	Abcam	Cat. # ab32815
Mouse monoclonal anti-CI-MPR antibody	Abcam	Cat. # ab2733
Mouse monoclonal anti-LAMP1 antibody	DSHB	Cat. # H4A3
Mouse monoclonal anti-GST antibody	Proteintech	Cat. # 66001-2-Ig
Rabbit polyclonal anti-TagRFP antibody	Thermo Fisher	Cat. # R10367
Mouse monoclonal anti-HA antibody	Enzo Life Sciences	Cat. # ENZ-ABS290
Mouse monoclonal anti-PI4P antibody	Echelon Biosciences	Cat. # Z-P004
Mouse monoclonal anti-PI(4,5)P_2 antibody	Echelon Biosciences	Cat. # Z-P045
Mouse monoclonal anti-Cathepsin D antibody	BD	Cat. # 610800
Mouse monoclonal anti-a-tubulin antibody	Sigma	Cat. # T9026
Rabbit polyclonal anti-LC3 antibody	MBL	Cat. # PM036
Mouse monoclonal anti-WIPI2	Abcam	Cat. # ab105459
HRP-conjugated anti-mouse antibody	Sigma	Cat. # GENA931
HRP-conjugated anti-rabbit antibody	Sigma	Cat. # GENA934
Alexa Fluor 488 goat anti-mouse IgG (H+L)	Invitrogen	Cat. # A-11029
Alexa Fluor 488 goat anti-rabbit IgG (H+L)	Invitrogen	Cat. # A-11034
Alexa Fluor 488 goat anti-mouse IgM	Invitrogen	Cat. # A-21042
Alexa Fluor 555 goat anti-mouse IgG (H+L)	Invitrogen	Cat. # A-21424
Alexa Fluor 555 goat anti-rabbit IgG (H+L)	Invitrogen	Cat. # A-41429
Alexa Fluor 633 goat anti-mouse IgM	Invitrogen	Cat. # A-21046
Alexa Fluor 633 goat anti-rabbit IgG (H+L)	Invitrogen	Cat. # A-21071
Alexa Fluor 647 goat anti-mouse IgG (H+L)	Invitrogen	Cat. # A-21236
Bacterial and virus strains		
Escherichia Coli: BL21 (DE3)	(New England Biolabs)	Cat. # C2527I

Chemicals, peptides, and recombinant proteins		
1,2-di-(9Z-octadecenoyl)-*sn*-glycero-3-phosphocholine (DOPC)	Avanti Polar Lipids	Cat. # 850375
1,2-di-(9Z-octadecenoyl)-*sn*-glycero-3-phosphoethanolamine (DOPE)	Avanti Polar Lipids	Cat. # 850725
L-α-phosphatidylinositol-4-phosphate (PI4P)	Avanti Polar Lipids	Cat. # 840045
N-[12-[(7-nitro-2-1,3-benzoxadiazol-4-yl)amino]dodecanoyl]-sphingosine-1-phosphocholine (NBD-C12-SM)	Avanti Polar Lipids	Cat. # 810219
1-oleoyl-2-[6-[(7-nitro-2-1,3-benzoxadiazol-4-yl)amino]hexanoyl]-sn-glycero-3-phosphocholine (NBD-C6-PC)	Avanti Polar Lipids	Cat. # 810130
Cholesterol	Sigma	Cat. # C8667
Polyvinyl alcohol Mw~145000	Sigma	Cat. # 814894
OSW-1	Cyman Chemicals	Cat. # 30310
Bafilomycin A	SignalChem Lifesciences	Cat. # V115-905-100
Pepstatin A, BODIPY FL conjugate	Thermo Fisher	Cat. # P12271
LysoTracker Deep Red	Thermo Fisher	Cat. # L12492
L-Leucyl-L-Leucine methyl ester	MedChemExpress	Cat. # 129905A
Lovastatin	Cayman Chemical	Cat. # 10010338
Mevaloic acid	Sigma	Cat. # 90469
Lipoprotein deficient serum	Sigma	Cat. # S5394-50ML
ProLong Diamond Antifade Mountant	Thermo Ficher	Cat. # P36970
jetPRIME transfection reagent	Ozyme	Cat. # POL114-07
Lipofectamine RNAiMAX	Thermo Ficher	Cat. # 13778
Protease inhibitor cocktail	Sigma	Cat. # 539139
Critical commercial assays		
Membrane Lipid Strip	Echelon Biosciences	P-6002
Experimental models: Cell lines		
HeLa (wild-type) cells	ATCC	CCL-2
GGA1 knockout (GGA1KO) HeLa cells	(*14*)	RKEN BRC cell bank RCB5659

mCat#8 (control) HeLa cells	(*19, 20*)	N/A
SMS1/2 double knockout (DKO) HeLa cells	(*19, 20*)	N/A
Oligonucleotides		
GGA1 siRNA#1: ON-TARGETplus Human GGA1 siRNA	Horizon Discovery	L-013694-00-0005
GGA1 siRNA#2: Silencer Select GGA1 siRNA	FISHER SCIENTIFIC	4392420
Recombinant DNA		
pEGFP-GGA1	This paper	N/A
pEGFP-GGA2	(*97*)	N/A
pEGFP-GGA3	(*98*)	N/A
pmCherry-GGA1	This paper	N/A
pEGFP-VHS	This paper	N/A
pEGFP-GAT	This paper	N/A
pEGFP-GAE	This paper	N/A
pmCherry-GAT	This paper	N/A
pNT-EqtII-mKate	(*21*)	N/A
pNT-EqtII-mClover3	This paper	N/A
pNT-EqtII-EGFP	This paper	N/A
pEGFP-NT-Lys	This paper	N/A
pmCherry-NT-Lys	This paper	N/A
pEGFP-D4H	This paper	N/A
pEGFP-PH-OSBP	(*42*)	N/A
pcDNA3/hARF1-HA	Addgene	#79409
pcDNA3/mARF1 Q71L-HA	Addgene	#79410
pcDNA3/mARF1 T31N-HA	Addgene	#79411
pEGFP-GGA1-bSMaseC	This paper	N/A
pEGFP-GGA1-bSMaseC D321A/H322A	This paper	N/A
pEGFP-LC3	Addgene	#21073
pEGFP-bSMaseC-LC3	This paper	N/A
pEGFP-GGA1 R260A	This paper	N/A
pGEX6p-1-GST-GAT	This paper	N/A

pGEX6p-1-GAT	This paper	N/A
pGEX6p-1-sfGFP-D4H	(41)	N/A
APEX2-GBP	(96)	N/A
EGFP-PA-APEX2-ChBP	(96)	N/A
pEGFP-TECPR1	(67)	N/A
Str-KDEL-SS-SBP-MMP11-EGFP	This paper	N/A
NT-EqtII-mKate-P2A-mTagBFP2	This paper	N/A
mTagBFP2-P2A-mCherry-NT-Lys	This paper	N/A
mTagBFP2-P2A-mEGFP-D4H	This paper	N/A
Software and algorithms		
FIJI: an open-source platform for biological image analysis	National Institute of Health	https://imagej.net/software/fiji/downloads
AlphaFold server	Google Deepmind	https://alphafoldserver.com

LIST OF MOVIES

Movie S1. Live-cell imaging of WT HeLa cells doubly labeled with GFP-GGA1 and mKate-β1,4-galactosyltransferase. (**A**) WT HeLa cells transfected with GFP-GGA1. Live-cell imaging was performed 24 h post-transfection at 10 s intervals for 2.5 min. (**B**) WT HeLa cells co-transfected with GFP-GGA1 and mKate-β1,4-galactosyltransferase. Images were acquired at 15 s intervals for 5 min.

Movie S2. The GAT domain induces tubulation of GUVs. Time-lapse imaging of GUVs composed of DOPC:NBD-SM (99:1) without **(A)** or with **(B)** GAT, and DOPC:DOPE:PI4P:Chol:NBD-SM (14:70:5:10:1) without **(C)** or with **(D)** GAT. Images were acquired every 0.5 s for 30 s.

Movie S3. The GAT domain induces intracellular tubulation SM-rich membranes. Wild-type HeLa cells transfected with GFP-GAT and NT-EqtII-mKate. Live-cell images of GFP-GAT **(A)** and NT-EqtII-mKate **(B)** were captured 24 h post-transfection at 15 s intervals for 5 min.

Movie S4. The GAT domain induces tubulation of GUVs in the absence of Chol. Time-lapse imaging of GUVs composed of DOPC:DOPE:PI4P:NBD-SM (24:70:5:1) in the presence of GAT. Images were captured at 0.5 s intervals for 30 s.

Movie S5. The GAT domain induces intracellular tubulation of Chol-rich membranes. WT HeLa cells transfected with GFP-D4H and mCherry-GAT. Live-cell imaging of GFP-D4H was performed 24 h post-transfection at 2 s intervals for 2 min.

Movie S6. 3D animation of AlphaFold 3-predicted trimeric GAT domain structure.

Movie S7. Live cell imaging of WT and GGA1 KO HeLa cells labeled with Str-KDEL-SS-SBP-MMP11-EGFP. WT (**A**) and GGA1 KO cells (**B**) were transfected with Str-KDEL-SS-SBP-MMP11-EGFP and treated with 40 µM biotin. Secretion of the MMP11 cargo from ER-Golgi was imaged at 5 min interval for 100 min.

Movie S8. Live cell imaging of WT HeLa cells doubly labeled with GFP-GGA1 and mCherry-NT-Lys. WT HeLa cells co-transfected with GFP-GGA1 and mCherry-NT-Lys. Live-cell images of GFP-GGA1 (green) and mCherry-NT-Lys (magenta) were acquired at 10 s intervals for 20 min.

Movie S9. Live cell imaging of WT HeLa cells triply labeled with GFP-GGA1, NT-EqtII-mKate and LysoTracker. WT HeLa cells co-transfected with GFP-GGA1 and NT-EqtII-mKate and treated with LysoTracker Deep Red during the final 30 min. Live-cell images of GFP-GGA1 (green), NT-EqtII-mKate (magenta), and LysoTracker (blue) were acquired at 10 s intervals for 20 min.

REFERENCES

1. T. Kobayashi, A. K. Menon, Transbilayer lipid asymmetry. *Curr. Biol.* **28**, R386–R391 (2018).

2. M. Doktorova, J. L. Symons, I. Levental, Structural and functional consequences of reversible lipid asymmetry in living membranes. *Nat. Chem. Biol.* **16**, 1321–1330 (2020).

3. J. C. Holthuis, A. K. Menon, Lipid landscapes and pipelines in membrane homeostasis. *Nature* **510**, 48–57 (2014).

4. T. G. Pomorski, A. K. Menon, Lipid somersaults: Uncovering the mechanisms of protein-mediated lipid flipping. *Prog. Lipid Res.* **64**, 69–84 (2016).

5. H. W. Shin, H. Takatsu, Substrates of P4-ATPases: Beyond aminophospholipids (phosphatidylserine and phosphatidylethanolamine). *FASEB J.* **33**, 3087–3096 (2019).

6. Y. Lange, M. H. Swaisgood, B. V. Ramos, T. L. Steck, Plasma membranes contain half the phospholipid and 90% of the cholesterol and sphingomyelin in cultured human fibroblasts. *J. Biol. Chem.* **264**, 3786–3793 (1989).

7. A. J. Verkleij, R. F. Zwaal, B. Roelofsen, P. Comfurius, D. Kastelijn, L. L. van Deenen, The asymmetric distribution of phospholipids in the human red cell membrane. A combined study using phospholipases and freeze-etch electron microscopy. *Biochim. Biophys. Acta* **323**, 178–193 (1973).

8. R. J. Clarke, K. R. Hossain, K. Cao, Physiological roles of transverse lipid asymmetry of animal membranes. *Biochim. Biophys. Acta* **1862**, 183382 (2020).

9. T. Kobayashi, Mapping transmembrane distribution of sphingomyelin. *Emerg. Top. Life Sci.* **7**, 31–45 (2023).

10. F. G. Tafesse, P. Ternes, J. C. Holthuis, The multigenic sphingomyelin synthase family. *J. Biol. Chem.* **281**, 29421–29425 (2006).

11. S. Mitsutake, K. Zama, H. Yokota, T. Yoshida, M. Tanaka, M. Mitsui, M. Ikawa, M. Okabe, Y. Tanaka, T. Yamashita, H. Takemoto, T. Okazaki, K. Watanabe, Y. Igarashi, Dynamic modification of sphingomyelin in lipid microdomains controls development of obesity, fatty liver, and type 2 diabetes. *J. Biol. Chem.* **286**, 28544–28555 (2011).

12. Y. A. Hannun, L. M. Obeid, Sphingolipids and their metabolism in physiology and disease. *Nat. Rev. Mol. Cell Biol.* **19**, 175–191 (2018).

13. M. Murate, M. Abe, K. Kasahara, K. Iwabuchi, M. Umeda, T. Kobayashi, Transbilayer distribution of lipids at nano scale. *J. Cell Sci.* **128**, 1627–1638 (2015).

14. M. Abe, A. Makino, M. Murate, F. Hullin-Matsuda, M. Yanagawa, Y. Sako, T. Kobayashi, PMP2/FABP8 induces PI(4,5)P2-dependent transbilayer reorganization of sphingomyelin in the plasma membrane. *Cell Rep.* **37**, 109935 (2021).

15. J. S. Bonifacino, The GGA proteins: Adaptors on the move. *Nat. Rev. Mol. Cell Biol.* **5**, 23–32 (2004).

16. T. Uemura, S. Waguri, Emerging roles of Golgi/endosome-localizing monomeric clathrin adaptors GGAs. *Anat. Sci. Int.* **95**, 12–21 (2020).

17. I. Meras, J. Maes, S. Lefrancois, Mechanisms regulating the sorting of soluble lysosomal proteins. *Biosci. Rep.* **42**, BSR20211856 (2022).

18. C. A. Lamb, T. Yoshimori, S. A. Tooze, The autophagosome: Origins unknown, biogenesis complex. *Nat. Rev. Mol. Cell Biol.* **14**, 759–774 (2013).

19. Y. Tachida, K. Kumagai, S. Sakai, S. Ando, T. Yamaji, K. Hanada, Chlamydia trachomatis-infected human cells convert ceramide to sphingomyelin without sphingomyelin synthases 1 and 2. *FEBS Lett.* **594**, 519–529 (2020).

20. T. Yamaji, K. Nishikawa, K. Hanada, Transmembrane BAX inhibitor motif containing (TMBIM) family proteins perturbs a trans-Golgi network enzyme, Gb3 synthase, and reduces Gb3 biosynthesis. *J. Biol. Chem.* **285**, 35505–35518 (2010).

21. T. Mori, T. Niki, Y. Uchida, K. Mukai, Y. Kuchitsu, T. Kishimoto, S. Sakai, A. Makino, T. Kobayashi, H. Arai, Y. Yokota, T. Taguchi, K. G. N. Suzuki, A non-toxic equinatoxin-II reveals the dynamics and distribution of sphingomyelin in the cytosolic leaflet of the plasma membrane. *Sci. Rep.* **14**, 16872 (2024).

22. E. Kiyokawa, T. Baba, N. Otsuka, A. Makino, S. Ohno, T. Kobayashi, Spatial and functional heterogeneity of sphingolipid-rich membrane domains. *J. Biol. Chem.* **280**, 24072–24084 (2005).

23. R. Ishitsuka, A. Yamaji-Hasegawa, A. Makino, Y. Hirabayashi, T. Kobayashi, A lipid-specific toxin reveals heterogeneity of sphingomyelin-containing membranes. *Biophys. J.* **86**, 296–307 (2004).

24. A. Makino, M. Abe, M. Murate, T. Inaba, N. Yilmaz, F. Hullin-Matsuda, T. Kishimoto, N. L. Schieber, T. Taguchi, H. Arai, G. Anderluh, R. G. Parton, T. Kobayashi, Visualization of the heterogeneous membrane distribution of sphingomyelin associated with cytokinesis, cell polarity, and sphingolipidosis. *FASEB J.* **29**, 477–493 (2015).

25. B. Bakrac, A. Kladnik, P. Macek, G. McHaffie, A. Werner, J. H. Lakey, G. Anderluh, A toxin-based probe reveals cytoplasmic exposure of Golgi sphingomyelin. *J. Biol. Chem.* **285**, 22186–22195 (2010).

26. A. L. Szymczak, C. J. Workman, Y. Wang, K. M. Vignali, S. Dilioglou, E. F. Vanin, D. A. Vignali, Correction of multi-gene deficiency in vivo using a single 'self-cleaving' 2A peptide-based retroviral vector. *Nat. Biotechnol.* **22**, 589–594 (2004).

27. I. Iacovache, M. Bischofberger, F. G. van der Goot, Structure and assembly of pore-forming proteins. *Curr. Opin. Struct. Biol.* **20**, 241–246 (2010).

28. U. Ros, A. J. Garcia-Saez, More than a pore: The interplay of pore-forming proteins and lipid membranes. *J. Membr. Biol.* **248**, 545–561 (2015).

29. D. Lingwood, K. Simons, Lipid rafts as a membrane-organizing principle. *Science* **327**, 46–50 (2010).

30. I. Levental, K. R. Levental, F. A. Heberle, Lipid rafts: Controversies resolved, mysteries remain. *Trends Cell Biol.* **30**, 341–353 (2020).

31. P. Niekamp, F. Scharte, T. Sokoya, L. Vittadello, Y. Kim, Y. Deng, E. Sudhoff, A. Hilderink, M. Imlau, C. J. Clarke, M. Hensel, C. G. Burd, J. C. M. Holthuis, Ca^{2+} activated sphingomyelin scrambling and turnover mediate ESCRT-independent lysosomal repair. *Nat. Commun.* **13**, 1875 (2022).

32. M. Uhlen, L. Fagerberg, B. M. Hallstrom, C. Lindskog, P. Oksvold, A. Mardinoglu, A. Sivertsson, C. Kampf, E. Sjostedt, A. Asplund, I. Olsson, K. Edlund, E. Lundberg, S. Navani, C. A. Szigyarto, J. Odeberg, D. Djureinovic, J. O. Takanen, S. Hober, T. Alm, P. H. Edqvist, H. Berling, H. Tegel, J. Mulder, J. Rockberg, P. Nilsson, J. M. Schwenk, M. Hamsten, K. von Feilitzen, M. Forsberg, L. Persson, F. Johansson, M. Zwahlen, G. von Heijne, J. Nielsen, F. Ponten, Proteomics. Tissue-based map of the human proteome. *Science* **347**, 1260419 (2015).

33. P. Ghosh, J. Griffith, H. J. Geuze, S. Kornfeld, Mammalian GGAs act together to sort mannose 6-phosphate receptors. *J. Cell Biol.* **163**, 755–766 (2003).

34. J. Wang, H. Q. Sun, E. Macia, T. Kirchhausen, H. Watson, J. S. Bonifacino, H. L. Yin, PI4P promotes the recruitment of the GGA adaptor proteins to the trans-Golgi network and regulates their recognition of the ubiquitin sorting signal. *Mol. Biol. Cell* **18**, 2646–2655 (2007).

35. J. C. McIntyre, R. G. Sleight, Fluorescence assay for phospholipid membrane asymmetry. *Biochemistry* **30**, 11819–11827 (1991).

36. S. Vehring, L. Pakkiri, A. Schroer, N. Alder-Baerens, A. Herrmann, A. K. Menon, T. Pomorski, Flip-flop of fluorescently labeled phospholipids in proteoliposomes reconstituted with Saccharomyces cerevisiae microsomal proteins. *Eukaryot. Cell* **6**, 1625–1634 (2007).

37. T. Kobayashi, R. E. Pagano, ATP-dependent fusion of liposomes with the Golgi apparatus of perforated cells. *Cell* **55**, 797–805 (1988).

38. R. Ishitsuka, T. Saito, H. Osada, Y. Ohno-Iwashita, T. Kobayashi, Fluorescence image screening for chemical compounds modifying cholesterol metabolism and distribution. *J. Lipid Res.* **52**, 2084–2094 (2011).

39. Y. Shimada, M. Maruya, S. Iwashita, Y. Ohno-Iwashita, The C-terminal domain of perfringolysin O is an essential cholesterol-binding unit targeting to cholesterol-rich microdomains. *Eur. J. Biochem.* **269**, 6195–6203 (2002).

40. B. B. Johnson, P. C. Moe, D. Wang, K. Rossi, B. L. Trigatti, A. P. Heuck, Modifications in perfringolysin O domain 4 alter the cholesterol concentration threshold required for binding. *Biochemistry* **51**, 3373–3382 (2012).

41. K. Takahashi, K. Kanerva, L. Vanharanta, L. Almeida-Souza, D. Lietha, V. M. Olkkonen, E. Ikonen, ORP2 couples LDL-cholesterol transport to FAK activation by endosomal cholesterol/PI(4,5)P_2 exchange. *EMBO J.* **40**, e106871 (2021).

42. B. Mesmin, J. Bigay, J. Moser von Filseck, S. Lacas-Gervais, G. Drin, B. Antonny, A four-step cycle driven by PI(4)P hydrolysis directs sterol/PI(4)P exchange by the ER-Golgi tether OSBP. *Cell* **155**, 830–843 (2013).

43. V. M. Olkkonen, E. Ikonen, Getting to grips with the oxysterol-binding protein family - A forty year perspective. *Contact* **7**, 25152564241273598 (2024).

44. A. W. Burgett, T. B. Poulsen, K. Wangkanont, D. R. Anderson, C. Kikuchi, K. Shimada, S. Okubo, K. C. Fortner, Y. Mimaki, M. Kuroda, J. P. Murphy, D. J. Schwalb, E. C. Petrella, I. Cornella-Taracido, M. Schirle, J. A. Tallarico, M. D. Shair, Natural products reveal cancer cell dependence on oxysterol-binding proteins. *Nat. Chem. Biol.* **7**, 639–647 (2011).

45. J. Abramson, J. Adler, J. Dunger, R. Evans, T. Green, A. Pritzel, O. Ronneberger, L. Willmore, A. J. Ballard, J. Bambrick, S. W. Bodenstein, D. A. Evans, C. C. Hung, M. O'Neill, D. Reiman, K. Tunyasuvunakool, Z. Wu, A. Zemgulyte, E. Arvaniti, C. Beattie, O. Bertolli, A. Bridgland, A. Cherepanov, M. Congreve, A. I. Cowen-Rivers, A. Cowie, M. Figurnov, F. B. Fuchs, H. Gladman, R. Jain, Y. A. Khan, C. M. R. Low, K. Perlin, A. Potapenko, P. Savy, S. Singh, A. Stecula, A. Thillaisundaram, C. Tong, S. Yakneen, E. D. Zhong, M. Zielinski, A.

Zidek, V. Bapst, P. Kohli, M. Jaderberg, D. Hassabis, J. M. Jumper, Accurate structure prediction of biomolecular interactions with AlphaFold 3. *Nature* **630**, 493–500 (2024).

46. T. Shiba, M. Kawasaki, H. Takatsu, T. Nogi, N. Matsugaki, N. Igarashi, M. Suzuki, R. Kato, K. Nakayama, S. Wakatsuki, Molecular mechanism of membrane recruitment of GGA by ARF in lysosomal protein transport. *Nat. Struct. Biol.* **10**, 386–393 (2003).

47. J. Li, Z. Gao, D. Zhao, L. Zhang, X. Qiao, Y. Zhao, H. Ding, P. Zhang, J. Lu, J. Liu, H. Jiang, C. Luo, C. Chen, PI-273, a substrate-competitive, specific small-molecule inhibitor of PI4KIIalpha, inhibits the growth of breast cancer cells. *Cancer Res.* **77**, 6253–6266 (2017).

48. C. Wiedemann, T. Schafer, M. M. Burger, Chromaffin granule-associated phosphatidylinositol 4-kinase activity is required for stimulated secretion. *EMBO J.* **15**, 2094–2101 (1996).

49. A. L. Boman, C. Zhang, X. Zhu, R. A. Kahn, A family of ADP-ribosylation factor effectors that can alter membrane transport through the trans-Golgi. *Mol. Biol. Cell* **11**, 1241–1255 (2000).

50. C. S. Chen, W. N. Chen, M. Zhou, S. Arttamangkul, R. P. Haugland, Probing the cathepsin D using a BODIPY FL-pepstatin A: Applications in fluorescence polarization and microscopy. *J. Biochem. Biophys. Methods* **42**, 137–151 (2000).

51. J. F. Presley, N. B. Cole, T. A. Schroer, K. Hirschberg, K. J. Zaal, J. Lippincott-Schwartz, ER-to-Golgi transport visualized in living cells. *Nature* **389**, 81–85 (1997).

52. K. Nishimoto-Morita, H. W. Shin, H. Mitsuhashi, M. Kitamura, Q. Zhang, L. Johannes, K. Nakayama, Differential effects of depletion of ARL1 and ARFRP1 on membrane trafficking between the trans-Golgi network and endosomes. *J. Biol. Chem.* **284**, 10583–10592 (2009).

53. G. Boncompain, S. Divoux, N. Gareil, H. de Forges, A. Lescure, L. Latreche, V. Mercanti, F. Jollivet, G. Raposo, F. Perez, Synchronization of secretory protein traffic in populations of cells. *Nat. Methods* **9**, 493–498 (2012).

54. E. R. Motrescu, M. C. Rio, Cancer cells, adipocytes and matrix metalloproteinase 11: A vicious tumor progression cycle. *Biol. Chem.* **389**, 1037–1041 (2008).

55. W. Sakamoto, D. Canals, S. Salamone, J. Allopenna, C. J. Clarke, J. Snider, L. M. Obeid, Y. A. Hannun, Probing compartment-specific sphingolipids with targeted bacterial sphingomyelinases and ceramidases. *J. Lipid Res.* **60**, 1841–1850 (2019).

56. R. Puertollano, R. C. Aguilar, I. Gorshkova, R. J. Crouch, J. S. Bonifacino, Sorting of mannose 6-phosphate receptors mediated by the GGAs. *Science* **292**, 1712–1716 (2001).

57. T. F. Martin, PI(4,5)P(2) regulation of surface membrane traffic. *Curr. Opin. Cell Biol.* **13**, 493–499 (2001).

58. D. H. Jones, J. B. Morris, C. P. Morgan, H. Kondo, R. F. Irvine, S. Cockcroft, Type I phosphatidylinositol 4-phosphate 5-kinase directly interacts with ADP-ribosylation factor 1 and is responsible for phosphatidylinositol 4,5-bisphosphate synthesis in the golgi compartment. *J. Biol. Chem.* **275**, 13962–13966 (2000).

59. N. S. Roy, X. Jian, O. Soubias, P. Zhai, J. R. Hall, J. N. Dagher, N. P. Coussens, L. M. Jenkins, R. Luo, I. O. Akpan, M. D. Hall, R. A. Byrd, M. E. Yohe, P. A. Randazzo, Interaction of the N terminus of ADP-ribosylation factor with the PH domain of the GTPase-activating protein ASAP1 requires phosphatidylinositol 4,5-bisphosphate. *J. Biol. Chem.* **294**, 17354–17370 (2019).

60. G. R. Hammond, G. Schiavo, R. F. Irvine, Immunocytochemical techniques reveal multiple, distinct cellular pools of PtdIns4P and PtdIns(4,5)P_2. *Biochem. J.* **422**, 23–35 (2009).

61. K. S. Matlin, K. Simons, Reduced temperature prevents transfer of a membrane glycoprotein to the cell surface but does not prevent terminal glycosylation. *Cell* **34**, 233–243 (1983).

62. T. Kobayashi, M. H. Beuchat, J. Chevallier, A. Makino, N. Mayran, J. M. Escola, C. Lebrand, P. Cosson, J. Gruenberg, Separation and characterization of late endosomal membrane domains. *J. Biol. Chem.* **277**, 32157–32164 (2002).

63. J. Collette, J. P. Bocock, K. Ahn, R. L. Chapman, G. Godbold, S. Yeyeodu, A. H. Erickson, Biosynthesis and alternate targeting of the lysosomal cysteine protease cathepsin L. *Int. Rev. Cytol.* **241**, 1–51 (2004).

64. Y. Kabeya, N. Mizushima, T. Ueno, A. Yamamoto, T. Kirisako, T. Noda, E. Kominami, Y. Ohsumi, T. Yoshimori, LC3, a mammalian homologue of yeast Apg8p, is localized in autophagosome membranes after processing. *EMBO J.* **19**, 5720–5728 (2000).

65. H. C. Dooley, M. Razi, H. E. Polson, S. E. Girardin, M. I. Wilson, S. A. Tooze, WIPI2 links LC3 conjugation with PI3P, autophagosome formation, and pathogen clearance by recruiting Atg12-5-16L1. *Mol. Cell* **55**, 238–252 (2014).

66. A. Yamamoto, Y. Tagawa, T. Yoshimori, Y. Moriyama, R. Masaki, Y. Tashiro, Bafilomycin A1 prevents maturation of autophagic vacuoles by inhibiting fusion between autophagosomes and lysosomes in rat hepatoma cell line, H-4-II-E cells. *Cell Struct. Func.* **23**, 33–42 (1998).

67. L. Wetzel, S. Blanchard, S. Rama, V. Beier, A. Kaufmann, T. Wollert, TECPR1 promotes aggrephagy by direct recruitment of LC3C autophagosomes to lysosomes. *Nat. Commun.* **11**, 2993 (2020).

68. K. B. Boyle, C. J. Ellison, P. R. Elliott, M. Schuschnig, K. Grimes, M. S. Dionne, C. Sasakawa, S. Munro, S. Martens, F. Randow, TECPR1 conjugates LC3 to damaged endomembranes upon detection of sphingomyelin exposure. *EMBO J.* **42**, e113012 (2023).

69. N. Kaur, L. R. de la Ballina, H. S. Haukaas, M. L. Torgersen, M. Radulovic, M. J. Munson, A. Sabirsh, H. Stenmark, A. Simonsen, S. R. Carlsson, A. H. Lystad, TECPR1 is activated by damage-induced sphingomyelin exposure to mediate noncanonical autophagy. *EMBO J.* **42**, e113105 (2023).

70. Y. Hayashi, Y. Nemoto-Sasaki, N. Matsumoto, T. Tanikawa, S. Oka, Y. Tanaka, S. Arai, I. Wada, T. Sugiura, A. Yamashita, Carboxyl-terminal tail-mediated homodimerizations of sphingomyelin synthases are responsible for efficient export from the endoplasmic reticulum. *J. Biol. Chem.* **292**, 1122–1141 (2017).

71. K. Hu, Q. Zhang, Y. Chen, J. Yang, Y. Xia, B. Rao, S. Li, Y. Shen, M. Cao, H. Lu, A. Qin, X. C. Jiang, D. Yao, J. Zhao, L. Zhou, Y. Cao, Cryo-EM structure of human sphingomyelin synthase and its mechanistic implications for sphingomyelin synthesis. *Nat. Struct. Mol. Biol.* **31**, 884–895 (2024).

72. R. X. Gu, S. Baoukina, D. P. Tieleman, Cholesterol flip-flop in heterogeneous membranes. *J. Chem. Theory Comput.* **15**, 2064–2070 (2019).

73. S. Baral, I. Levental, E. Lyman, Composition dependence of cholesterol flip-flop rates in physiological mixtures. *Chem. Physics Lipids* **232**, 104967 (2020).

74. J. M. Duran, F. Campelo, J. van Galen, T. Sachsenheimer, J. Sot, M. V. Egorov, C. Rentero, C. Enrich, R. S. Polishchuk, F. M. Goni, B. Brugger, F. Wieland, V. Malhotra, Sphingomyelin organization is required for vesicle biogenesis at the Golgi complex. *EMBO J.* **31**, 4535–4546 (2012).

75. I. Menon, T. Sych, Y. Son, T. Morizumi, J. Lee, O. P. Ernst, G. Khelashvili, E. Sezgin, J. Levitz, A. K. Menon, A cholesterol switch controls phospholipid scrambling by G protein-coupled receptors. *J. Biol. Chem.* **300**, 105649 (2024).

76. S. Suer, S. Misra, L. F. Saidi, J. H. Hurley, Structure of the GAT domain of human GGA1: A syntaxin amino-terminal domain fold in an endosomal trafficking adaptor. *Proc. Natl. Acad. Sci. U.S.A.* **100**, 4451–4456 (2003).

77. M. Langer, D. Langosch, Is lipid flippase activity of SNARE transmembrane domains required for membrane fusion? *FEBS Lett.* **585**, 1021–1024 (2011).

78. B. J. Peter, H. M. Kent, I. G. Mills, Y. Vallis, P. J. Butler, P. R. Evans, H. T. McMahon, BAR domains as sensors of membrane curvature: The amphiphysin BAR structure. *Science* **303**, 495–499 (2004).

79. J. C. Dawson, J. A. Legg, L. M. Machesky, Bar domain proteins: A role in tubulation, scission and actin assembly in clathrin-mediated endocytosis. *Trends Cell Biol.* **16**, 493–498 (2006).

80. S. Ruskamo, R. P. Yadav, S. Sharma, M. Lehtimaki, S. Laulumaa, S. Aggarwal, M. Simons, J. Burck, A. S. Ulrich, A. H. Juffer, I. Kursula, P. Kursula, Atomic resolution view into the structure-function relationships of the human myelin peripheral membrane protein P2. *Acta Crystallogr.* **70**, 165–176 (2014).

81. A. Mizuike, S. Sakai, K. Katoh, T. Yamaji, K. Hanada, The C10orf76-PI4KB axis orchestrates CERT-mediated ceramide trafficking to the distal Golgi. *J. Cell Biol.* **222**, e202111069 (2023).

82. M. Krauss, J. Y. Jia, A. Roux, R. Beck, F. T. Wieland, P. De Camilli, V. Haucke, Arf1-GTP-induced tubule formation suggests a function of Arf family proteins in curvature acquisition at sites of vesicle budding. *J. Biol. Chem.* **283**, 27717–27723 (2008).

83. M. Liljedahl, Y. Maeda, A. Colanzi, I. Ayala, J. Van Lint, V. Malhotra, Protein kinase D regulates the fission of cell surface destined transport carriers from the trans-Golgi network. *Cell* **104**, 409–420 (2001).

84. F. Campelo, J. van Galen, G. Turacchio, S. Parashuraman, M. M. Kozlov, M. F. Garcia-Parajo, V. Malhotra, Sphingomyelin metabolism controls the shape and function of the Golgi cisternae. *eLife* **6**, e24603 (2017).

85. R. Lundmark, G. J. Doherty, Y. Vallis, B. J. Peter, H. T. McMahon, Arf family GTP loading is activated by, and generates, positive membrane curvature. *Biochem. J.* **414**, 189–194 (2008).

86. M. Lokar, D. Kabaso, N. Resnik, K. Sepcic, V. Kralj-Iglic, P. Veranic, R. Zorec, A. Iglic, The role of cholesterol-sphingomyelin membrane nanodomains in the stability of intercellular membrane nanotubes. *Int. J. Nanomedicine* **7**, 1891–1902 (2012).

87. P. Crottet, D. M. Meyer, J. Rohrer, M. Spiess, ARF1.GTP, tyrosine-based signals, and phosphatidylinositol 4,5-bisphosphate constitute a minimal machinery to recruit the AP-1 clathrin adaptor to membranes. *Mol. Biol. Cell* **13**, 3672–3682 (2002).

88. D. Schmitt, S. Bozkurt, P. Henning-Domres, H. Huesmann, S. Eimer, L. Bindila, C. Behrends, E. Boyle, F. Wilfling, G. Tascher, C. Munch, C. Behl, A. Kern, Lipid and protein content profiling of isolated native autophagic vesicles. *EMBO Rep.* **23**, e53065 (2022).

89. K. Matoba, T. Kotani, A. Tsutsumi, T. Tsuji, T. Mori, D. Noshiro, Y. Sugita, N. Nomura, S. Iwata, Y. Ohsumi, T. Fujimoto, H. Nakatogawa, M. Kikkawa, N. N. Noda, Atg9 is a lipid scramblase that mediates autophagosomal membrane expansion. *Nat. Struct. Mol. Biol.* **27**, 1185–1193 (2020).

90. D. Isola, Z. Elazar, Phospholipid supply for autophagosome biogenesis. *J.Mol. Biol.* **436**, 168691 (2024).

91. E. G. Bligh, W. J. Dyer, A rapid method of total lipid extraction and purification. *Can. J. Biochem. Physiol.* **37**, 911–917 (1959).

92. A. Weinberger, F. C. Tsai, G. H. Koenderink, T. F. Schmidt, R. Itri, W. Meier, T. Schmatko, A. Schroder, C. Marques, Gel-assisted formation of giant unilamellar vesicles. *Biophys. J.* **105**, 154–164 (2013).

93. L. P. Wilhelm, C. Wendling, B. Vedie, T. Kobayashi, M. P. Chenard, C. Tomasetto, G. Drin, F. Alpy, STARD3 mediates endoplasmic reticulum-to-endosome cholesterol transport at membrane contact sites. *EMBO J.* **36**, 1412–1433 (2017).

94. A. Punjani, J. Rubinstein, D. Fleet, M. A. Brubaker, cryoSPARC: Algorithms for rapid unsupervised cryo-EM structure determination. *Nat. Methods* **14**, 290–296 (2017).

95. E. C. Meng, T. D. Goddard, E. F. Pettersen, G. S. Couch, Z. J. Pearson, J. H. Morris, T. E. Ferrin, UCSF ChimeraX: Tools for structure building and analysis. *Prot. Sci.* **32**, e4792 (2023).

96. N. Ariotti, T. E. Hall, J. Rae, C. Ferguson, K. A. McMahon, N. Martel, R. E. Webb, R. I. Webb, R. D. Teasdale, R. G. Parton, Modular detection of GFP-labeled proteins for rapid screening by electron microscopy in cells and organisms. *Dev. Cell* **35**, 513–525 (2015).

97. T. Uemura, S. Kametaka, S. Waguri, GGA2 interacts with EGFR cytoplasmic domain to stabilize the receptor expression and promote cell growth. *Sci. Rep.* **8**, 1368 (2018).

98. M. Wakasugi, S. Waguri, S. Kametaka, Y. Tomiyama, S. Kanamori, Y. Shiba, K. Nakayama, Y. Uchiyama, Predominant expression of the short form of GGA3 in human cell lines and tissues. *Biochem. Biophys. Res. Commun.* **306**, 687–692 (2003).
